# Composition-Dependent Electrochemical Performance of Anatase TiO_2_/Polyaniline–Polythiophene Hybrid Electrodes for Supercapacitor Applications

**DOI:** 10.3390/nano16211442

**Published:** 2026-11-07

**Authors:** Suria Shaheen, Mansoor Ahmad, Khalid Talha, Alamgir

**Affiliations:** 1 Institute of Chemical Science, University of Peshawar, Peshawar 25120, Pakistan; surishaheen111@gmail.com; 2 Innovation Center of Yangtze River Delta, Zhejiang University, Jiaxing 314100, China; mansoor@zju.edu.cn; 3 Institution of Analytical Chemistry, Department of Chemistry, Zhejiang University, Hangzhou 310058, China; 4 Department of Chemistry, University of Bari Aldo Moro, Via Edoardo Orabona 4, 70125 Bari, Italy; 5 Centre for Optical and Electromagnetic Research, National Engineering Research Center for Optical Instruments, College of Optical Science and Engineering, Zhejiang University, Hangzhou 310058, China

**Keywords:** TiO_2_, conducting polymer nanohybrids, polyaniline/polythiophene, interface engineering, pseudocapacitive energy storage

## Abstract

Conducting polymer-based hybrid materials have attracted considerable attention for electrochemical energy storage applications; however, their practical performance remains limited by poor structural stability, sluggish charge transport, and degradation during repeated redox cycling. Herein, a ${\mathrm{TiO}}_{2}$-integrated polyaniline/polythiophene (PANI/PTh) nanohybrid was designed through an inverse emulsion polymerization strategy to construct a synergistic polymer-metal oxide interface for enhanced electrochemical performance. The successful formation of the copolymer (CoP)/${\mathrm{TiO}}_{2}$ hybrid was confirmed through spectroscopic, structural, and morphological analyses, including Fourier-transform infrared spectroscopy (FTIR), X-ray diffraction (XRD), scanning electron microscopy (SEM), and energy-dispersive X-ray spectroscopy (EDX). The incorporation of anatase ${\mathrm{TiO}}_{2}$ nanoparticles into the conducting polymer framework promoted interfacial charge transfer, improved electrolyte accessibility, and enhanced the reversible redox activity of the polymer matrix. Electrochemical investigations using cyclic voltammetry (CV), galvanostatic charge-discharge (GCD), and electrochemical impedance spectroscopy (EIS) demonstrated the superior capacitive behavior of the optimized ${\mathrm{CoP}/\mathrm{TiO}}_{2}$ composite compared with individual polymer and ${\mathrm{TiO}}_{2}$ components. The optimized electrode delivered a specific capacitance of 433 F $g^{-1}$ from CV measurements and 414 F $g^{-1}$ from GCD analysis at 0.75 A $g^{-1}$, together with an energy density of 9.4 Wh ${\mathrm{kg}}^{-1}$ and a power density of 175 W ${\mathrm{kg}}^{-1}$. Cycling stability measurements revealed that the optimized ${\mathrm{CoP}/\mathrm{TiO}}_{2}$-5 electrode retained approximately 61% of its initial capacitance after 1000 consecutive cycles, indicating appreciable capacitance degradation during repeated electrochemical cycling. Although the hybrid exhibited enhanced initial charge-storage performance, further improvements in cycling stability are required for long-term supercapacitor applications. The incorporation of anatase ${\mathrm{TiO}}_{2}$ into the conducting polymer matrix resulted in composition-dependent structural and electrochemical characteristics, with the optimized ${\mathrm{CoP}/\mathrm{TiO}}_{2}$ formulation exhibiting enhanced charge-storage performance relative to the investigated reference materials.

## 1. Introduction

The increasing global demand for energy, driven by rapid industrialization and population growth, has intensified the need for efficient and sustainable energy technologies. Consequently, significant research efforts have been directed toward the development of renewable energy systems and advanced energy storage and conversion devices, including batteries, fuel cells, and supercapacitors, to enable efficient energy utilization and address emerging energy challenges. In recent years, supercapacitors (SCs) have attracted increasing research attention owing to their high-power density, rapid charge-discharge capability, and extended cycling stability compared with conventional rechargeable batteries. These advantages make SCs promising candidates for applications requiring efficient energy storage and high-performance power delivery [1,2]. Additional advantages of SCs include rapid charge-discharge capability, a simple operating mechanism, high power delivery efficiency, and low maintenance requirements, making them attractive for a wide range of energy storage applications [3,4,5].

Charge-storage mechanisms in SCs are generally classified into two categories: electric double-layer capacitors (EDLCs) and pseudocapacitors (PCs). In EDLCs, energy storage occurs through electrostatic charge separation at the electrode-electrolyte interface, resulting in the formation of an electric double layer without involving charge-transfer reactions. In contrast, pseudocapacitive storage involves fast and reversible faradaic redox reactions between the electrode material and electrolyte species, enabling charge transfer across the interface and providing higher capacitance compared with EDLC-based systems [4,6]. The electrochemical performance of SCs is strongly influenced by the intrinsic properties of electrode materials, including their electrical conductivity, surface area, pore structure, pore-size distribution, and electrochemical activity. These parameters govern electrolyte ion accessibility, charge-transfer kinetics, and the overall energy-storage capability of supercapacitor electrodes [7]. The development of electrode materials with high surface area, suitable pore structures, and efficient charge-transfer pathways remains a key strategy for enhancing the performance of SCs. Graphene oxide/PANI hybrid electrodes have also been evaluated for supercapacitor applications [8]. Carbon-based materials are widely employed as electrode materials for EDLCs due to their high specific surface area, excellent electrical conductivity, chemical stability, and ability to facilitate rapid ion adsorption at the electrode-electrolyte interface [5,9,10]. In contrast, transition metal oxides and noble metal oxides, such as NiO, ${\mathrm{IrO}}_{2}$, and ${\mathrm{MnO}}_{2}$, along with conducting polymers (CPs), are widely utilized as electrode materials for pseudocapacitive supercapacitors due to their reversible faradaic redox reactions and high theoretical capacitance [11,12,13]. In EDLCs, charge storage primarily occurs through electrostatic adsorption of ions at the electrode-electrolyte interface, whereas pseudocapacitive materials utilize reversible faradaic reactions throughout the active electrode matrix, enabling greater charge storage capability. Consequently, pseudocapacitive electrodes generally exhibit higher specific capacitance than conventional EDLC materials due to their bulk redox-active nature. To enhance pseudocapacitive performance, conducting polymers such as polypyrrole (PPy), polyaniline (PANI), and polythiophene (PTh), as well as their derivatives, have been widely combined with metal oxides to construct hybrid electrode materials with improved electrical conductivity, structural stability, and electrochemical activity [14,15,16,17,18]. More broadly, incorporating inorganic phases into polymer matrices, including polyaniline-containing hybrids, is an established route to tuning the structural, electronic, and functional properties of polymer nanocomposites [19,20].

Conducting polymers (CPs) have emerged as promising electrode materials for pseudocapacitive energy storage owing to their high electrical conductivity, environmental stability, mechanical flexibility, low density, and cost-effectiveness compared with many conventional metal oxide materials. Moreover, their facile synthesis, tunable chemical structure, and compatibility with various nanomaterials enable the fabrication of advanced hybrid electrode architectures with improved electrochemical performance [21]. Among various conducting polymers, polyaniline (PANI) and its derivatives have attracted considerable attention due to their good environmental stability, tunable electrical conductivity, reversible redox activity, and favorable electrochemical properties, making them promising candidates for pseudocapacitive electrode applications [22]. Despite these advantages, PANI suffers from limited cycling stability due to structural degradation, volume expansion/contraction during repeated doping-dedoping processes, and gradual loss of electrochemical activity during long-term charge-discharge cycles [23,24]. Polythiophene (PTh) is another promising conducting polymer for electrochemical energy storage owing to its conjugated molecular structure, reversible redox activity, chemical stability, and tunable electrical properties. Previous studies have demonstrated that its electrochemical performance, including specific capacitance, conductivity, and cycling stability, is strongly influenced by polymerization conditions, doping state, and surface morphology [23,25,26].

Recent investigations have further demonstrated the potential of conducting polymer–metal oxide composites for electrochemical energy storage applications. Hacinecipoğlu et al. [27] developed poly(aniline-co-thiophene)/PbO composite electrodes for supercapacitor applications, reporting a specific capacitance of 294 F $g^{-1}$ at a scan rate of 10 mV $s^{-1}$ and capacitance retention of 70.69% after 1000 cycles. Similarly, Fazal et al. [28] synthesized ${\mathrm{TiO}}_{2}$/PANI composites through in situ chemical oxidative polymerization and achieved a specific capacity of 925 C $g^{-1}$ at 1 A $g^{-1}$, with 98% capacitance retention after 5000 cycles for the corresponding asymmetric supercapacitor device. More recently, Kumar et al. [29] investigated ${\mathrm{PTh/MoO}}_{3}{\mathrm{/TiO}}_{2}$ ternary nanocomposites, obtaining a specific capacitance of 271.3 F $g^{-1}$ at 1 A $g^{-1}$ and demonstrating the potential of metal oxide incorporation in PTh-based electrode architectures. These studies highlight the continued development of conducting polymer–metal oxide hybrids for improving electrochemical energy storage. However, differences in polymer composition, oxide selection, synthesis conditions, and electrochemical testing configurations influence the reported performance. Consequently, systematic evaluation of the electrochemical behavior of PANI/PTh–anatase ${\mathrm{TiO}}_{2}$ formulations relative to their individual components remains relevant for understanding composition-dependent charge-storage characteristics.

Nevertheless, the charge-storage performance of pristine PTh may be limited by inefficient charge transport, restricted accessibility of electrochemically active sites, and structural changes during repeated doping–dedoping processes. To overcome these limitations, researchers have investigated the incorporation of complementary materials, including graphene derivatives and metal oxides, to improve electrical conductivity, electrochemical reversibility, and specific capacitance [30,31,32]. These findings highlight the importance of compositional modification and hybrid electrode architectures for enhancing the electrochemical performance of PTh-based supercapacitor electrodes [30]. However, PTh generally exhibits lower specific capacitance, limited electrical conductivity, and reduced power output compared with other conducting polymers, which restricts its practical application as a standalone electrode material for high-performance supercapacitors [33]. Therefore, the practical application of pristine PANI and PTh electrodes in supercapacitors is limited by their inherent drawbacks, including structural instability, restricted charge transport, and insufficient long-term cycling performance. However, conducting polymers generally suffer from structural instability during repeated charge-discharge cycles due to mechanical deformation, volumetric changes, and degradation of the polymer backbone during redox processes. Among the metal oxides explored for conducting-polymer electrodes, titanium dioxide (${\mathrm{TiO}}_{2}$) is of interest because of its chemical stability, low cost, and compatibility with polymer-based composite preparation [34]. Its relatively low intrinsic electronic conductivity, however, limits the performance of unmodified ${\mathrm{TiO}}_{2}$ electrodes. Previous studies have addressed this limitation through ${\mathrm{PANI}/\mathrm{TiO}}_{2}$ architectures, including PANI nanowires within titania nanotube arrays [15], surfactant-assisted ${\mathrm{PANI}/\mathrm{TiO}}_{2}$ composites [17], and grafted PANI/titania nanotube electrodes [18]. Xie et al. [15], for example, reported a specific capacitance of 732 F $g^{-1}$ at 1 A $g^{-1}$ for a PANI–titania nanotube electrode. The use of inverse emulsion polymerization for preparing ${\mathrm{PANI}/\mathrm{TiO}}_{2}$ nanocomposites has also been established [35]. ${\mathrm{Polythiophene/TiO}}_{2}$ electrodes have similarly been examined [16,36]; Thakur and Choudhary [36] reported 250 F $g^{-1}$ at 1 A $g^{-1}$ for a ${\mathrm{PTh/TiO}}_{2}$ composite using a two-electrode configuration. A titanium-doped PTh-co-PANI system was previously investigated for photovoltaic applications [37], but that ${\mathrm{TiCl}}_{4}$-derived system is distinct from an anatase ${\mathrm{TiO}}_{2}$-containing supercapacitor electrode.

Taken together, these reports show that neither ${\mathrm{conducting-polymer/TiO}}_{2}$ hybridization nor inverse emulsion polymerization constitutes a new synthetic concept by itself. The question investigated here is more specific: how the electrochemical response of anatase ${\mathrm{TiO}}_{2}$-containing PANI/PTh formulations compares with the corresponding PANI, PTh, ${\mathrm{TiO}}_{2}$, ${\mathrm{PANI}/\mathrm{TiO}}_{2}$, and PANI/PTh (CoP) materials tested under the same laboratory conditions. Examining the ${\mathrm{CoP}/\mathrm{TiO}}_{2}$ formulation series alongside these controls provides a basis for describing changes in capacitive response and impedance behavior. However, previously reported capacitance values obtained with different electrode configurations, current densities, electrolytes, and mass normalizations cannot be used to establish a direct performance ranking without appropriate qualification.

Accordingly, this study prepares ${\mathrm{CoP}/\mathrm{TiO}}_{2}$ composites by inverse emulsion polymerization, keeping the aniline amount constant (0.2 mL) while jointly varying thiophene (0.1–0.9 mL) and anatase ${\mathrm{TiO}}_{2}$ (0.04–0.12 g) across five formulations. The products are examined by XRD, FTIR, UV–visible spectroscopy, SEM, and EDX, and their electrochemical behavior is evaluated using CV, GCD, and EIS in a three-electrode system. The contribution of this work is the comparative characterization of this ${\mathrm{mixed-polymer/TiO}}_{2}$ composition series and its measured capacitance relative to the included reference materials. Because the thiophene and ${\mathrm{TiO}}_{2}$ contents change simultaneously, the observed trends are interpreted as formulation-dependent behavior rather than evidence for an independent ${\mathrm{TiO}}_{2}$-loading effect or a uniquely established interfacial charge-transfer mechanism. Although ${\mathrm{PANI}/\mathrm{TiO}}_{2}$ and ${\mathrm{PTh/TiO}}_{2}$ composites have been extensively investigated for electrochemical energy storage, the influence of combining both conducting polymers with anatase ${\mathrm{TiO}}_{2}$ on the charge-storage characteristics of the resulting hybrid formulations remains an area of interest. Previous investigations have demonstrated the preparation of ${\mathrm{PANI}/\mathrm{TiO}}_{2}$ composites through inverse emulsion polymerization [35], as well as the electrochemical applications of ${\mathrm{PANI}/\mathrm{TiO}}_{2}$ and ${\mathrm{PTh/TiO}}_{2}$ binary systems [15,16,17,18,36]. Furthermore, titanium-containing poly(aniline-co-thiophene) materials have been reported for photovoltaic applications [37], although their composition and intended application differ from the present anatase ${\mathrm{TiO}}_{2}$-based supercapacitor electrodes. Building upon these studies, the present work investigates a series of PANI/PTh–TiO_2_ nanocomposites synthesized through inverse emulsion polymerization, with particular emphasis on the relationship between formulation composition, structural characteristics, and electrochemical charge-storage performance. The synthesized composites were systematically characterized using XRD, FTIR, UV–visible spectroscopy, SEM, and EDX, while their electrochemical properties were evaluated through cyclic voltammetry, galvanostatic charge–discharge, and electrochemical impedance spectroscopy. The electrochemical response of the hybrid formulations was compared with that of the individual components and the corresponding binary materials to assess their relative performance. As the thiophene and ${\mathrm{TiO}}_{2}$ contents were varied simultaneously, the resulting performance trends are discussed in terms of overall composition-dependent behavior rather than being attributed exclusively to ${\mathrm{TiO}}_{2}$ incorporation. This comparative investigation provides insight into the potential of PANI/PTh–anatase ${\mathrm{TiO}}_{2}$ hybrid formulations as electrode materials for electrochemical energy storage.

## 2. Materials and Methods

The materials and detailed synthesis procedures are discussed below, whereas the characterization of the synthesized materials are discussed in Appendix A.

### 2.1. Material and Chemicals

Thiophene, ${\mathrm{TiO}}_{2}$, analytical-grade aniline (AcrosOrganics, Geel, Belgium), acetone (99.5%; Sigma-Aldrich, St. Louis, MO, USA), chloroform (Sigma-Aldrich, St. Louis, MO, USA), hydrochloric acid (37%), sodium dodecyl sulfate (Merck, Darmstadt, Germany), and ammonium persulfate (APS; (${\mathrm{NH}}_{4}$)_2_$S_{2}O_{8}$, 99%; Riedel-de Haën, Seelze, Germany) were used as received from the suppliers.

### 2.2. Synthesis of PANI

Inverse emulsion polymerization technique was used for the synthesis of PANI. A 0.20 g of sodium dodecyl sulfate (SDS) and 0.2 mL aniline monomer were added to the initially taken 50 mL chloroform, under constant magnetic stirring. Afterward, 25 mL aqueous solution of HCl (0.01 M) and APS (0.01 M) were dropwise added to the reaction mixture. Subsequently, the mixture was mechanically stirred for 24 h. During the course of the reaction, a color change from grassy green to blue and then dark green was observed. Lastly, PANI was extracted and cleaned with acetone and deionized water before placing it in an oven at 60 °C for drying. The process was optimized by carrying out several trials and the finally synthesized PANI was used in different characterizations.

### 2.3. Synthesis of PTh

Like PANI, PTh was also synthesized by the inverse emulsion polymerization technique. 50 mL chloroform was taken in a 250 mL flask. Then 0.20 g of SDS was added to the flask, followed by the addition of 0.2 mL of thiophene monomer under constant magnetic stirring. Likewise, an aqueous solution of 25 mL HCl (0.01 M) and APS (0.01 M) was added to the mixture dropwise. To properly react, the mixture was placed under continuous mechanical stirring for 24 h. During reaction, a color change to dark brown was observed. Afterwards, the organic phase containing PTh was separated and cleaned with acetone and deionized water. The sample was placed at 60 °C overnight in an oven for drying. The finally obtained dried PTh was used in different characterizations. A few trials were carried out in order to obtain optimized oxidant, monomer, solvent and surfactant conditions.

### 2.4. Synthesis of Polyaniline/Polythiophene Composite (CoP)

Typically, the same experimental process as for PANI and PTh synthesis was followed for the preparation of CoP, starting with 50 mL of chloroform in a round-bottom flask (250 mL), followed by an addition of 0.20 g SDS, succeeded by adding 0.2 mL of thiophene and 0.2 mL of aniline monomer under steady magnetic stirring. Afterward, an aqueous solution of HCl (0.01 M) and APS (0.01 M) was added to the mixture in drops. The sample was then placed on a mechanical stirrer for 24 h. The color of the sample changed to dark green during the solution’s reaction. Subsequently, CoP was extracted and rigorously washed with acetone and deionized water. Eventually, the sample was kept in an oven at 60 °C overnight to dry. The dried sample was collected for characterization. Various experiments were carried out to obtain optimized oxidant, solvent, surfactant and monomer concentrations.

### 2.5. Synthesis of Polyaniline/Titanium Dioxide (${\mathrm{PANI}/\mathrm{TiO}}_{2}$) Composite

In a standard inverse emulsion polymerization process as adopted for the PANI synthesis, 50 mL chloroform was taken in a 250 mL round-bottom flask, followed by the addition of 0.2 g of SDS and 0.2 mL of aniline monomer under continuous magnetic stirring. Subsequently, 25 mL HCl (0.01 M) and APS (0.01 M) aqueous solutions were added dropwise to the resulting mixture. After waiting for about 20 min for instigation of the chemical reaction, 0.04 g water sonicated ${\mathrm{TiO}}_{2}$ was added to the respective mixture. The sample was then placed on a mechanical stirrer for 24 h. The color of the sample changed to dark green during the process. Finally, ${\mathrm{PANI}/\mathrm{TiO}}_{2}$ was collected and washed carefully with acetone and deionized water. Later, the sample was kept for drying in an oven at 60 °C for 24 h. Finally, the dried ${\mathrm{PANI}/\mathrm{TiO}}_{2}$ was collected and used for different characterizations. Various experiments were performed to obtain optimized surfactant, monomer, oxidant and solvent concentrations.

### 2.6. Synthesis of CoP/${\mathrm{TiO}}_{2}$ Composite

The synthesis of ${\mathrm{CoP}/\mathrm{TiO}}_{2}$ composite, adopting the inverse emulsion polymerization technique, starts by taking 50 mL of chloroform in a 250 mL flask. Next, 0.2 g of SDS surfactant was added under continuous magnetic stirring, followed by the addition of 0.1 mL and 0.2 mL of thiophene and aniline monomers, respectively. After that, drops of 25 mL aqueous solution of HCl (0.01 M) and APS (0.01 M) were added to the mixture. After waiting for 20 min, 0.04 g of water-sonicated ${\mathrm{TiO}}_{2}$ was added to the sample. The reaction was left on a mechanical stirrer for 24 h. The synthesized ${\mathrm{CoP}/\mathrm{TiO}}_{2}$ was collected and carefully cleaned with acetone and deionized water. Later, the sample was kept for drying in an oven at 60 °C for 24 h. Thereafter, the dried ${\mathrm{CoP}/\mathrm{TiO}}_{2}$ composite was collected and used for several characterizations. Various experiments were conducted to obtain the optimized values of surfactant, monomer, oxidant and solvent. The synthesized ${\mathrm{CoP}/\mathrm{TiO}}_{2}$ composites with different concentrations are presented in Table 1.

The ${\mathrm{CoP}/\mathrm{TiO}}_{2}$-1 to ${\mathrm{CoP}/\mathrm{TiO}}_{2}$-5 formulations were prepared by simultaneously varying the amounts of thiophene and ${\mathrm{TiO}}_{2}$ while maintaining a constant aniline content. Consequently, this series represents combined variations in polymer and oxide precursor composition rather than an independent investigation of ${\mathrm{TiO}}_{2}$ loading. The electrochemical performance trends are, therefore, interpreted in relation to the overall formulation composition.

## 3. Results and Discussion

The structural, morphological, and electrochemical properties of the synthesized polymer-based ${\mathrm{TiO}}_{2}$ nanohybrids were systematically investigated to understand the relationship between material composition, interfacial interactions, and electrochemical performance. Various characterization techniques, including X-ray diffraction (XRD), Fourier-transform infrared spectroscopy (FTIR), scanning electron microscopy (SEM), energy-dispersive X-ray spectroscopy (EDX), cyclic voltammetry (CV), galvanostatic charge-discharge (GCD), and electrochemical impedance spectroscopy (EIS), were employed to confirm the successful formation of the hybrid structures and evaluate their energy-storage behavior.

### 3.1. XRD Study

**Figure A1:**
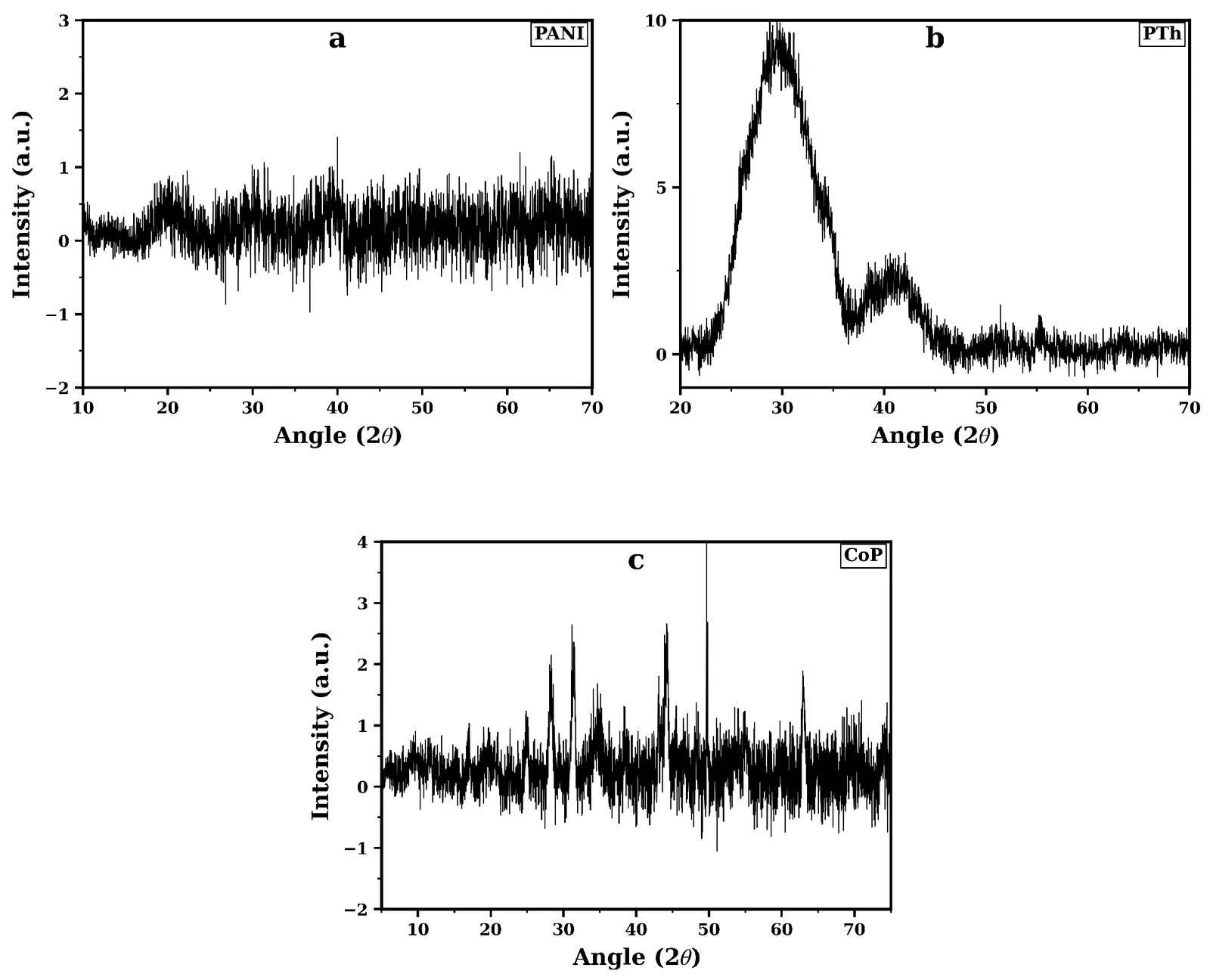
XRD pattern of (**a**) PANI, (**b**) PTh and (**c**) CoP-composite.

Figure A1 presents the X-ray diffraction (XRD) patterns of PANI, PTh, and the PANI/PTh copolymer (CoP). The XRD pattern of PANI (Figure A1a) exhibits broad features centered at $2\theta =20.4^{\circ }$ and $25.8^{\circ }$, consistent with short-range order in a largely disordered PANI sample. A PANI diffraction feature near $25^{\circ }$ has also been reported for chemically synthesized PANI [33].

The XRD pattern of PTh (Figure A1b) displays two broad diffraction regions located at 2θ=10–$20^{\circ }$ and 20–$35^{\circ }$, which are consistent with limited long-range order in the polymer [38]. The broad diffraction features indicate the absence of highly ordered crystalline domains, while the observed scattering behavior confirms the presence of short-range structural organization within the PTh matrix.

The XRD pattern of the PANI/PTh copolymer (Figure A1c) shows a broad continuous diffraction profile over the range of 2θ=10–$40^{\circ }$, incorporating the characteristic diffraction features of both PANI and PTh components. This behavior confirms the successful formation of the copolymer structure and indicates a predominantly amorphous/semi-crystalline nature. The overlap and broadening of diffraction peaks suggest the integration of PANI and PTh chains within a common polymer framework, while maintaining the characteristic conjugated structure of both conducting polymer components.

**Figure 1:**
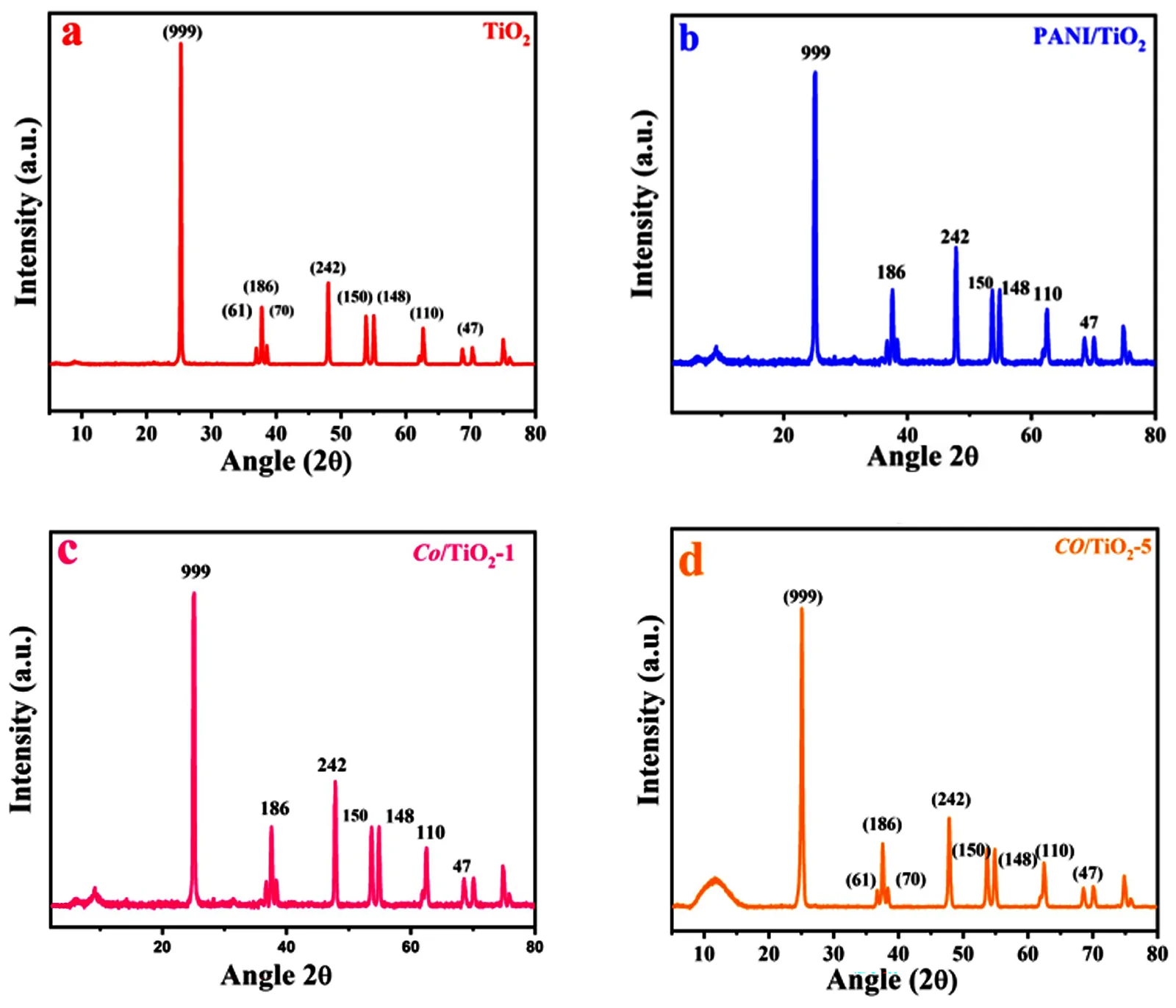
XRD patterns of (**a**) ${\mathrm{TiO}}_{2}$, (**b**) ${\mathrm{PANI}/\mathrm{TiO}}_{2}$, (**c**) ${\mathrm{CoP}/\mathrm{TiO}}_{2}$-1 and (**d**) ${\mathrm{CoP}/\mathrm{TiO}}_{2}$-5.

The XRD pattern of ${\mathrm{TiO}}_{2}$ (Figure 1a) shows pure anatase phase of ${\mathrm{TiO}}_{2}$. The ${\mathrm{TiO}}_{2}$ XRD peaks are in strong resemblance to the bulk ${\mathrm{TiO}}_{2}$, providing a tetragonal configuration with space group ${\mathrm{I4}}_{1}$/amd, and unit cell parameters a = b = 3.78 and c = 9.51. The reported peaks of anatase are well established, suggesting strong crystallinity in the sample. The peaks of anatase ${\mathrm{TiO}}_{2}$ partly appeared at $2\theta =25.3^{\circ }$ (999), $36.7^{\circ }$ (61), $37.8^{\circ }$ (186), $38.6^{\circ }$ (70), $48.0^{\circ }$ (242), $53.9^{\circ }$ (150), $55.8^{\circ }$ (148), $62.7^{\circ }$ (110) and $68.8^{\circ }$ (47).

XRD patterns of ${\mathrm{PANI}/\mathrm{TiO}}_{2}$ (Figure 1b) indicate that all the peaks corresponding to ${\mathrm{TiO}}_{2}$ anatase were present, indicating that the ${\mathrm{TiO}}_{2}$ state did not change during the polymerization cycle. However slight crystalline, tiny peaks occur at $2\theta =9.2^{\circ }$ [39], $15.0^{\circ }$ [40], which indicates PANI existence in the composite. On the other hand, in the nanocomposite ${\mathrm{PANI}/\mathrm{TiO}}_{2}$, a small decrease in peak strength was found for ${\mathrm{TiO}}_{2}$ peaks. This could be because of the interaction between the PANI backbone and ${\mathrm{TiO}}_{2}$ nanoparticles [40]. The ${\mathrm{CoP}/\mathrm{TiO}}_{2}$ pattern contains features near $2\theta =20^{\circ }$, $23^{\circ }$, and $25^{\circ }$ that overlap the broad polymer reflections and the strong anatase reflection near $25.3^{\circ }$; these overlapping features alone cannot independently identify both polymer units [33,41]. While the ${\mathrm{TiO}}_{2}$ XRD peaks resembling to card no (JCPDS No. 00-071-1166) providing a tetragonal configuration, were present in ${\mathrm{CoP}/\mathrm{TiO}}_{2}$ composite with small peak shifting at $2\theta =25.07^{\circ }$ (999), $37.28^{\circ }$ (61), $37.56^{\circ }$ (186), $38.37^{\circ }$ (70), $47.80^{\circ }$ (242), $53.67^{\circ }$ (150), $54.85^{\circ }$ (148), $62.50^{\circ }$ (110) and $68.51^{\circ }$ (47) [42], as shown in Figure 1c and Figure 1d, respectively.

### 3.2. Optical Studies

**Figure A2:**
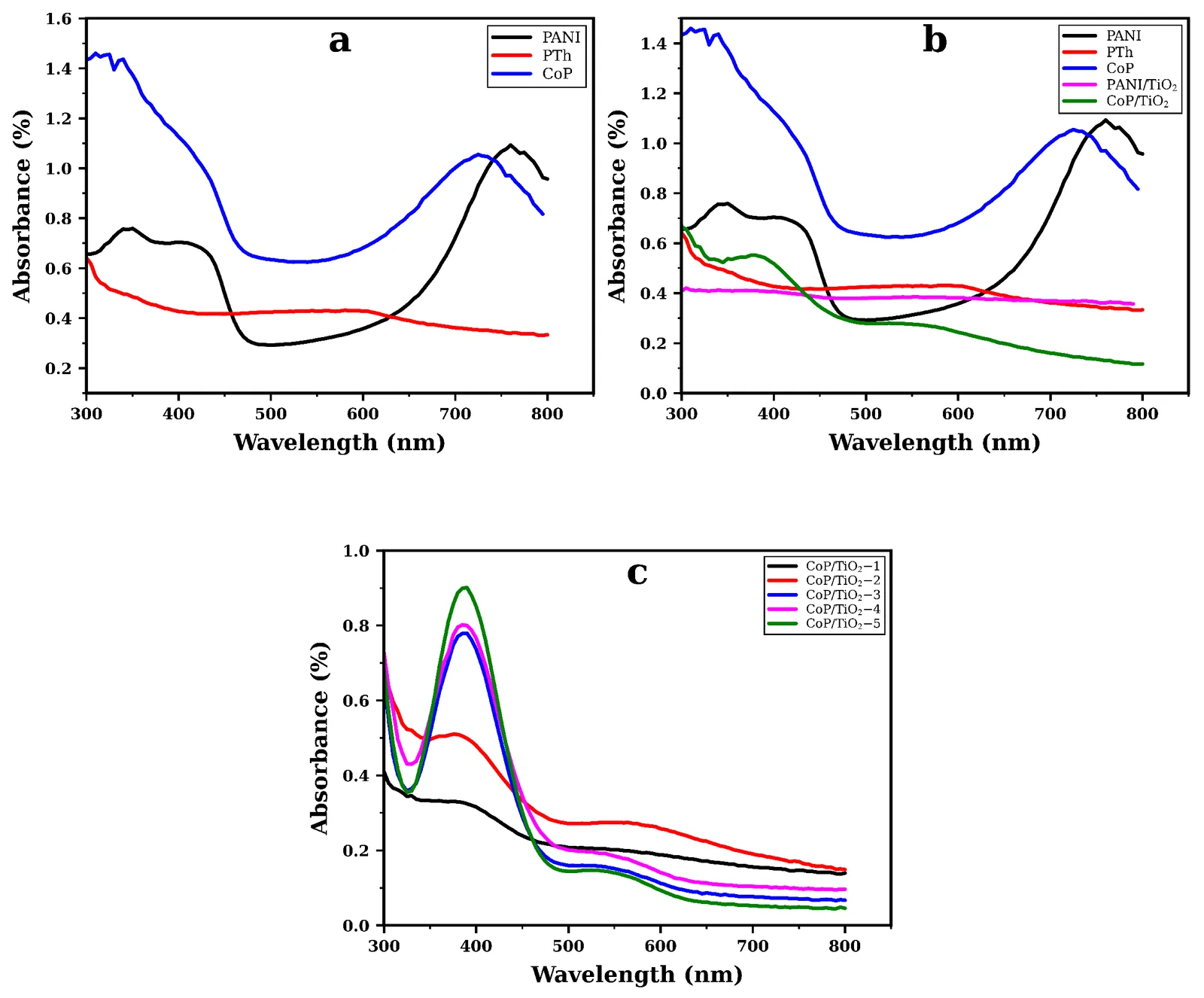
UV Visible spectra for (**a**) PANI, PTh, CoP (**b**) PANI, PTh, CoP, ${\mathrm{PANI}/\mathrm{TiO}}_{2}$, Composite (**c**) Composites ${\mathrm{CoP}/\mathrm{TiO}}_{2}$-1 to ${\mathrm{CoP}/\mathrm{TiO}}_{2}$-5.

The UV-Visible spectra of the synthesized materials were recorded in chloroform. The UV-Visible spectrum of ${\mathrm{TiO}}_{2}$ usually shows a steep absorption at around ∼390 nm [43,44]. Three absorption peaks around 345 nm, 415 nm and 760 nm were observed in the UV-Visible spectrum of PANI. The benzenoid ring π-$\pi^{*}$ transition is revealed by the peak at 345 nm [45]. The absorption peaks at 415 nm and 760 nm show polaronic and bipolaronic transitions [46]. In the spectrum of PTh, the absorption peak around 358 nm is associated with the π-$\pi^{*}$ transition [36,47]. The absorption for the aggregated molecules of PTh in a good solvent like chloroform occurs at around 602 nm [48]. The spectrum of CoP exhibits peaks between 300 and 345 nm and another peak at 730 nm. The 730 nm peak shows that the incorporation of thiophene units in the polyaniline has blue-shifted the 760 nm absorption peak of PANI. The absorption peaks are also shifted in the spectrum of ${\mathrm{PANI-TiO}}_{2}$ composites (Figure A2a) with respect to the PANI spectrum, indicating interaction between ${\mathrm{TiO}}_{2}$ particles and PANI chains [25,31].

In the UV-Vis spectrum of ${\mathrm{CoP}/\mathrm{TiO}}_{2}$ composite (Figure A2b), the peak around 545 nm is associated with the quinoid units of the polymer chain. The intense peak around 400 nm is attributed to the π-$\pi^{*}$ electronic transitions of the conjugated polymer backbones [37] as well as to the electronic transition of ${\mathrm{TiO}}_{2}$ [43,44]. Peak shifting shows the interaction of ${\mathrm{TiO}}_{2}$ with CoP and gives an indication of composite formation [25,49]. UV-Visible spectra of ${\mathrm{CoP}/\mathrm{TiO}}_{2}$ composites with varying concentrations (Figure A2c) depict identical features indicating the formation of composites of the same nature. However, the intensity of the peak around 400 nm increases and the peak intensity around 550 nm decreases with increasing concentration of ${\mathrm{TiO}}_{2}$ in the composites.

### 3.3. FTIR Spectroscopy

**Figure 2:**
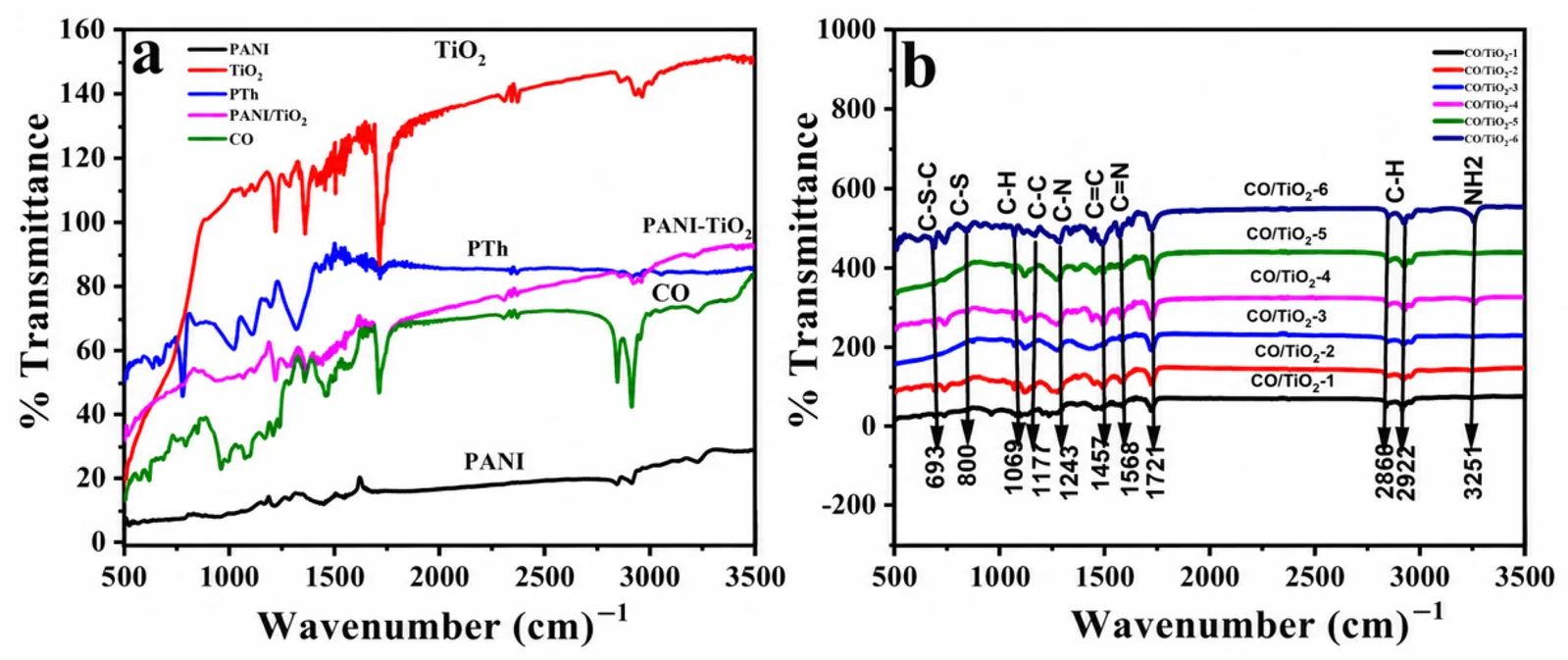
FTIR spectra of (**a**) PANI, PTh, *CoP*, ${\mathrm{TiO}}_{2}$, ${\mathrm{PANI}/\mathrm{TiO}}_{2}$, (**b**) *CoP*/${\mathrm{TiO}}_{2}$-1 to *CoP*/${\mathrm{TiO}}_{2}$-5.

FTIR spectra of all composites were recorded at room temperature by a spectrophotometer in the range of 400–$4000{\mathrm{cm}}^{-1}$. Figure 2a shows that the PANI spectrum has peaks at 832.5, 1171, 1219.8, 1290, 1452–1553, 2850, 2922, 3048.8 and $3239.3{\mathrm{cm}}^{-1}$. The band at $832.5{\mathrm{cm}}^{-1}$ is assigned to out of plane of C-H vibration of 1,4-disubstituted benzene ring [50]. The band at $1171{\mathrm{cm}}^{-1}$ is due to the N=Q=N stretching of PANI [51]. The peaks at $1553{\mathrm{cm}}^{-1}$ and $1452{\mathrm{cm}}^{-1}$ are attributed to the C=N and C=C stretching modes of the quinonoid and benzenoid rings, the bands at $1290{\mathrm{cm}}^{-1}$ and $1219{\mathrm{cm}}^{-1}$ are assigned to C-N stretching vibration for benzenoid units [52]. The band at $2850{\mathrm{cm}}^{-1}$ is for aliphatic hydrocarbon stretching vibration (${\mathrm{CH}}_{2}$, C-H) [53]. The peaks at 2922, 3048.8 and $3239.3{\mathrm{cm}}^{-1}$ show aromatic C-H stretching vibration [54], C=N stretching mode of vibration [55] and stretching vibrations of the secondary amine -NH- group in the spectrum of PANI, respectively [56].

The FTIR spectrum of PTh, Figure 2a, shows peaks at 675, 728, 782, 1021, 1117, 1427, 1643, 1721 and 2844–$3060{\mathrm{cm}}^{-1}$. The peak at $675{\mathrm{cm}}^{-1}$ is due to in-plane deformation of C-S-C of thiophene ring, while the peak at $782{\mathrm{cm}}^{-1}$ is assigned to C-S deformation [57]. Bending vibration of γ(C-H) in the aromatic ring occurs around $1021{\mathrm{cm}}^{-1}$ [58]. Several low-intensity peaks due to aromatic C-H stretching vibrations are present between 2844 and $3060{\mathrm{cm}}^{-1}$ [59]. The absorption band at $1643{\mathrm{cm}}^{-1}$ corresponds to the asymmetric stretching vibration of thiophene [60]. The peak at $1427{\mathrm{cm}}^{-1}$ was attributed to C=C asymmetric and symmetric stretching vibrations of thiophene units [26,61]. The PTh spectrum showed a very strong band attributed to the C-C vibration at $1117{\mathrm{cm}}^{-1}$ and a band at $728{\mathrm{cm}}^{-1}$ ascribed to the C-H out-of-plane vibration, which is very weak and indicates that the degree of polymerization of the prepared PTh is very high. The carbonyl group stretching vibration is found at $1721{\mathrm{cm}}^{-1}$ [55].

The spectrum of *CoP* in Figure 2a shows peaks at 63, 794.8, 848.8, 1069.8, 1171, 1219, 1458.8, 1571, 1701, 2844–$2916{\mathrm{cm}}^{-1}$ and $3233{\mathrm{cm}}^{-1}$. The peak at $630.3{\mathrm{cm}}^{-1}$ is assigned to C-S-C in-plane deformation, and the band at $794.8{\mathrm{cm}}^{-1}$ is ascribed to C-S bending of the thiophene ring. The peak at $848.8{\mathrm{cm}}^{-1}$ represents C-H out-of-plane vibration of PANI units. The band $1069.8{\mathrm{cm}}^{-1}$ is associated with γ (C-H) bending vibration and the band at $1171{\mathrm{cm}}^{-1}$ represents N=Q=N stretching vibration of PANI units in CO. The peak at $1219{\mathrm{cm}}^{-1}$ attributed to C-N stretching of benzenoid units. The peak at $1458.8{\mathrm{cm}}^{-1}$ shows symmetric and asymmetric stretching vibrations of C=C, and the band at $1571{\mathrm{cm}}^{-1}$ is assigned to the C=N stretching mode of benzenoid and quinoid rings. The bands at $1717{\mathrm{cm}}^{-1}$, 2844–$2916{\mathrm{cm}}^{-1}$ and $3233{\mathrm{cm}}^{-1}$ show carbonyl group stretching, C-H stretching vibration and vibration of hydrogen-bonded secondary amine (N-H), respectively. The presence of these slightly shifted peaks indicates the presence of both aniline and thiophene units in the as-prepared composite [43,44,45,46].

In the FTIR spectrum of ${\mathrm{TiO}}_{2}$ (Figure 2a), three peaks at 2964, 1364 and $1701{\mathrm{cm}}^{-1}$ are clearly visible. The peak at $2994{\mathrm{cm}}^{-1}$ is due to the stretching vibration of a weakly bonded hydroxyl group (O-H) due to moisture. The band at $1364{\mathrm{cm}}^{-1}$ is due to Ti-O vibration, and the band at $1701{\mathrm{cm}}^{-1}$ is attributed to the bending vibration of water in Ti-OH [32,56,62].

FTIR spectra, Figure 2b, of ${\mathrm{PANI}/\mathrm{TiO}}_{2}$ have characteristic bands at 1076, 1219, 1290, 1362.5, 2856.8, 2928, 3215.4 and $3412.7{\mathrm{cm}}^{-1}$. The band at $1076{\mathrm{cm}}^{-1}$ shows γ C-H bending vibration. Peaks at $1219{\mathrm{cm}}^{-1}$ and $1290{\mathrm{cm}}^{-1}$ represent C-N stretching vibrations of the benzenoid ring of PANI. The band at $1362.5{\mathrm{cm}}^{-1}$ indicates vibration of Ti-O. Peaks at 2856.8, $2928{\mathrm{cm}}^{-1}$, and 3215.4–$3412.7{\mathrm{cm}}^{-1}$ are attributed to aliphatic C-H, ${\mathrm{CH}}_{2}$ stretching vibrations and hydrogen-bonded secondary amines N-H stretching vibrations, respectively. The peak at $1715{\mathrm{cm}}^{-1}$ is assigned to the water bending vibration with ${\mathrm{TiO}}_{2}$ (Ti-OH) and the band at $2970{\mathrm{cm}}^{-1}$ is attributed to the weakly bonded hydroxyl group O-H with ${\mathrm{TiO}}_{2}$. The slight peak shifting is due to the interaction of ${\mathrm{TiO}}_{2}$ oxygen and PANI nitrogen atoms present in the prepared sample. All these peaks indicate the formation of binary composite of ${\mathrm{PANI}/\mathrm{TiO}}_{2}$ [25,46,47,50,51,63,64].

The spectrum of composite *CoP*/${\mathrm{TiO}}_{2}$, Figure 2b, gives bands at 693, 735, 795, 1079, 1123, 1219–1290, 1374, 1457, 1565, 3060, 1631, 1721, 2850–2922, and 3239–$3436{\mathrm{cm}}^{-1}$. Peaks at 693 and $795{\mathrm{cm}}^{-1}$ show the C-S-C and C-S deformation of the thiophene ring, while the peak at $735{\mathrm{cm}}^{-1}$ gives the out-of-plane vibration of C-H. The band at $1070{\mathrm{cm}}^{-1}$ is assigned for bending vibration of γ C-H. The band that represents C-C out of plane vibration, N=Q=N stretching vibration of PANI, and C-C stretching vibration of PANI are present at 1123, 1171 and 1219–$1290{\mathrm{cm}}^{-1}$, respectively. ${\mathrm{TiO}}_{2}$ presence in the composite is represented by the Ti-O peak at $1373{\mathrm{cm}}^{-1}$. Peaks at 1457 and $1565{\mathrm{cm}}^{-1}$ are assigned to the C=C symmetric and asymmetric stretching vibrations, while the band at $3060{\mathrm{cm}}^{-1}$ shows the C=N stretching vibration. The peak at $1631{\mathrm{cm}}^{-1}$ represents the asymmetric stretching vibration of the thiophene ring in the composite. Peaks at 1721, 2850–2922 and 3239–$3436{\mathrm{cm}}^{-1}$ designate carbonyl group stretching, C-H, ${\mathrm{CH}}_{2}$ stretching and N-H stretching vibrations of secondary amines, respectively. Figure 2b shows the FTIR spectra of *CoP*/${\mathrm{TiO}}_{2}$ composites of different compositions. Almost identical features are displayed by all spectra of the synthesized composites.

### 3.4. Morphological SEM Analysis

**Figure A3:**
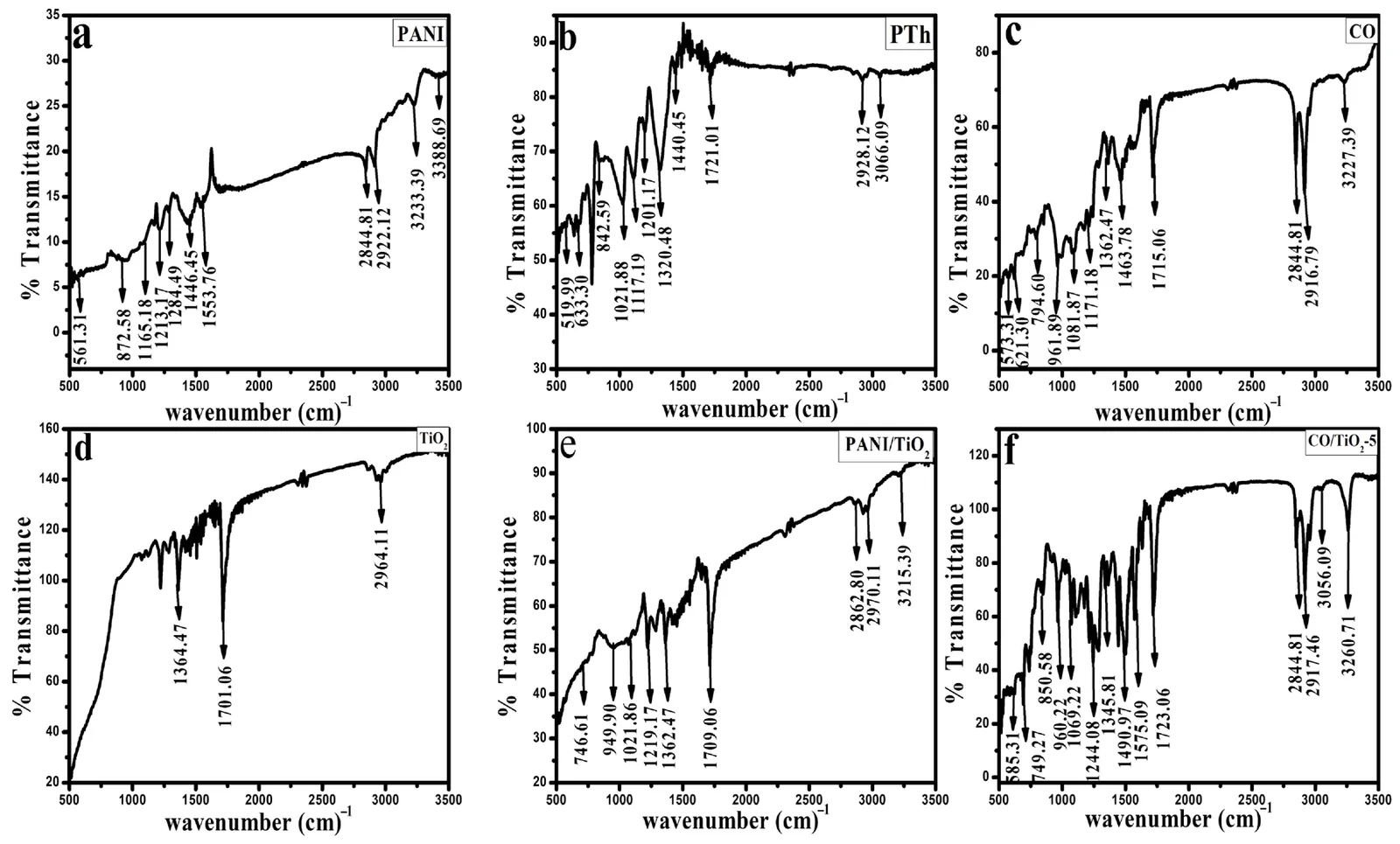
FTIR spectra of (**a**) PANI, (**b**) PTh, (**c**) *CoP* and (**d**) ${\mathrm{TiO}}_{2}$ (**e**) ${\mathrm{PANI}/\mathrm{TiO}}_{2}$, (**f**) ${\mathrm{CoP}/\mathrm{TiO}}_{2}$-5.

SEM is carried out to characterize the size and surface morphology of PANI, PTh, ${\mathrm{TiO}}_{2}$, ${\mathrm{PANI}/\mathrm{TiO}}_{2}$, CoP and ${\mathrm{CoP}/\mathrm{TiO}}_{2}$ composites. The SEM images of pure PANI, PTh, ${\mathrm{TiO}}_{2}$, ${\mathrm{PANI}/\mathrm{TiO}}_{2}$ and CoP are given in Figure A3. The surface morphology of PANI is determined by the conditions of synthesis. The SEM images show the most common morphology of PANI, which is granular and porous. Globular structure was used as a synonym for the granular form. The size of the grains is difficult to identify because the particles are aggregated, and it is difficult to distinguish the individual particles or grains from images.

Reaction conditions such as concentration of oxidant, monomer and surfactant and temperature play a major role in the synthesis of nanostructured composites. Both surfactant and oxidant have played a significant role in PTh morphology. It shows fibrous, honeycomb and porous morphology. The SEM image of the CoP polymer shows a tightly packed granular surface. SEM image of ${\mathrm{TiO}}_{2}$ shows spherical morphology with a uniform size distribution of nanoparticles, with 200 nm in size. The SEM image of the ${\mathrm{PANI}/\mathrm{TiO}}_{2}$ composite shows a globular structure with a rough and porous surface having small globules. The ${\mathrm{TiO}}_{2}$ nanoparticles are uniformly distributed in the agglomerate of the PANI porous matrix [36].

**Figure 3:**
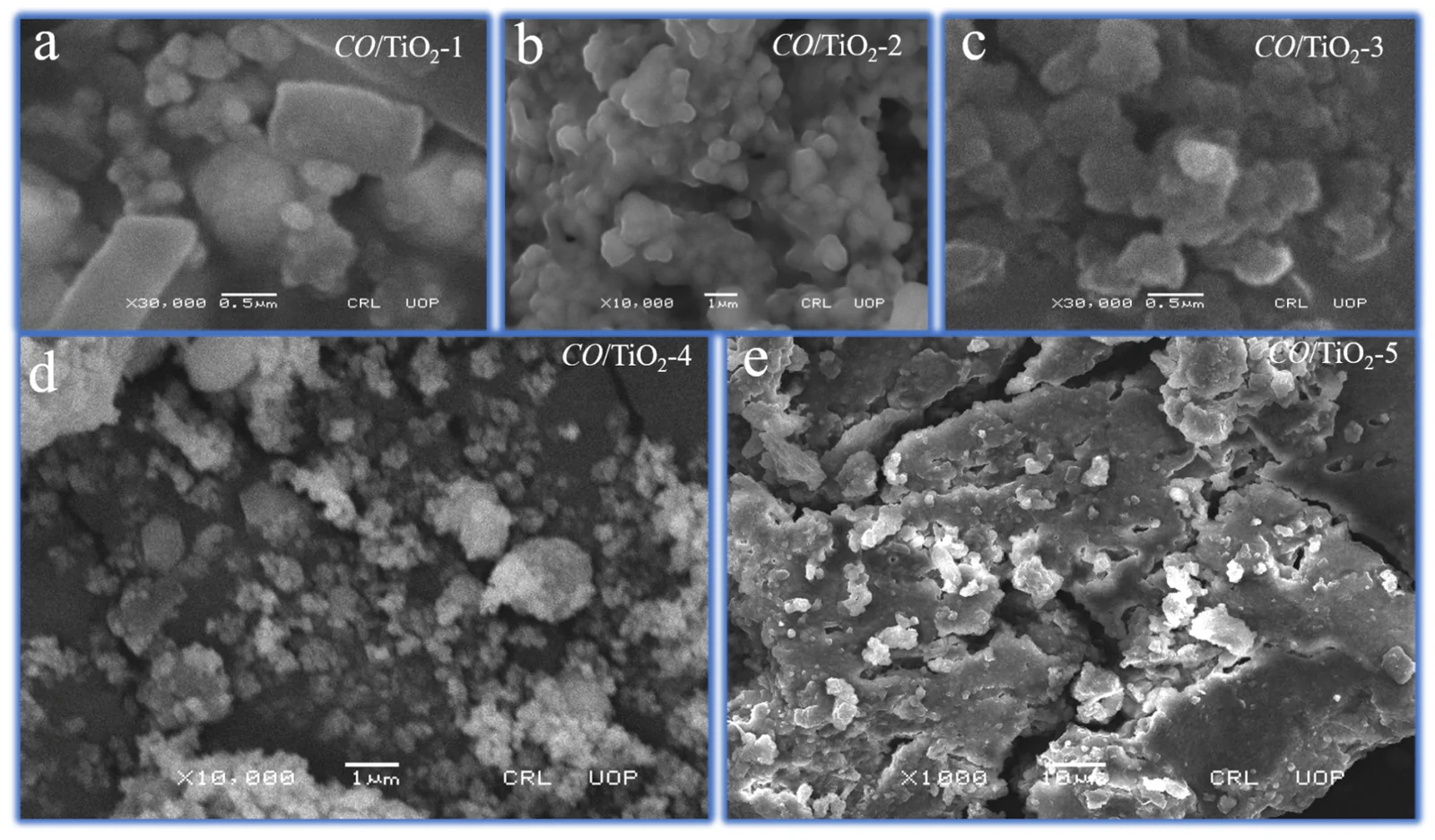
SEM images of (**a**–**e**) ${\mathrm{CoP}/\mathrm{TiO}}_{2}$-1 to ${\mathrm{CoP}/\mathrm{TiO}}_{2}$-5.

Figure 3a–e presents the SEM micrographs of ${\mathrm{CoP}/\mathrm{TiO}}_{2}$-1 to ${\mathrm{CoP}/\mathrm{TiO}}_{2}$-5, illustrating the morphological characteristics of the synthesized hybrid composites. The images reveal irregular granular structures and varying degrees of particle agglomeration, indicating differences in surface morphology among the investigated formulations. The observed aggregates suggest that the polymer matrix and ${\mathrm{TiO}}_{2}$ particles contribute to the overall morphological characteristics of the composites. However, the aggregation of particles makes it difficult to distinguish individual grains and accurately determine their dimensions. Although surface irregularities and interparticle spaces are visible in the SEM images, these observations do not provide quantitative information regarding specific surface area, pore volume, or pore-size distribution. Therefore, the SEM results are interpreted as qualitative evidence of morphological variation rather than direct confirmation of enhanced porosity or accessible surface area. Further characterization using nitrogen adsorption–desorption measurements would be necessary to quantitatively establish these textural properties.

### 3.5. EDX Analysis

**Figure A5:**
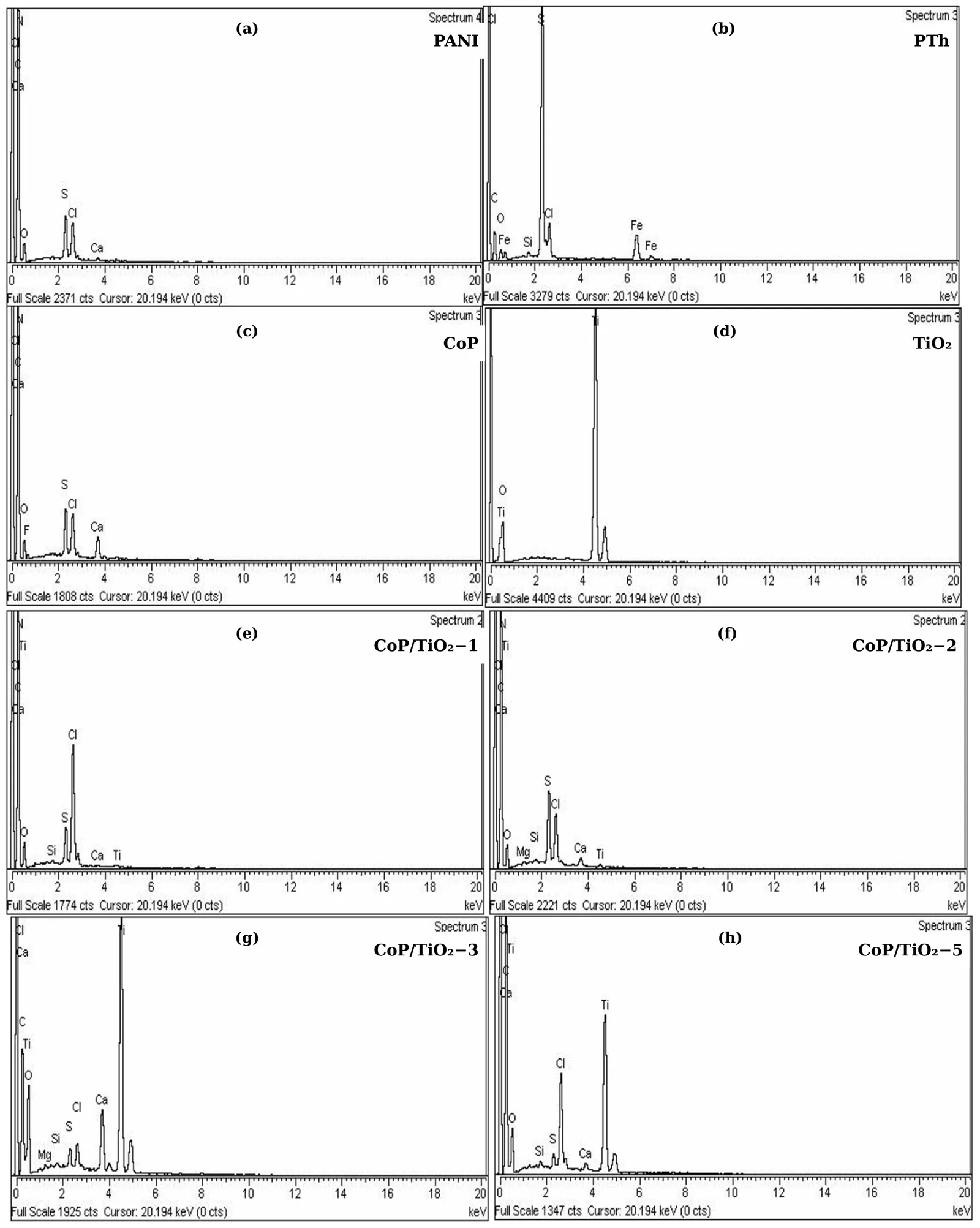
EDX spectra of PANI, PTh, CoP and ${\mathrm{TiO}}_{2}$. EDX spectra of ${\mathrm{CoP}/\mathrm{TiO}}_{2}$-1 to ${\mathrm{CoP}/\mathrm{TiO}}_{2}$-5 as indicated.

EDX spectra were recorded to gather information about the chemical composition of the prepared materials. It involves the contact of an X-ray excitation medium and the sample and is based on the concept that each product has a special atomic structure that enables the X-ray range to achieve specific peaks. Figure A5 shows the EDX spectra of the PANI, PTh, CoP and ${\mathrm{TiO}}_{2}$.The characteristic peaks in the PANI spectrum show the presence of various elements like C and N, in addition to the peaks of O and S. The presence of the main peaks of C and N suggests the formation of PANI. The pure PTh has the elemental composition of C, O, and S, along with Fe, Si and Cl impurities from the chemicals used. The EDX spectrum of ${\mathrm{TiO}}_{2}$ clearly indicates the presence of Ti and oxygen in the particles. The spectrum of CoP displays major peaks at C, N, S and O, indicating contributions from both PANI and PTh segments in the copolymer.

EDX spectra of ${\mathrm{CoP}/\mathrm{TiO}}_{2}$ composites with different compositions are shown in Figure A5. The presence of the characteristic peaks of PANI, PTh and ${\mathrm{TiO}}_{2}$ moieties is clearly observed in all spectra. The contribution of Ti is more prominent in the spectra of ${\mathrm{CoP}/\mathrm{TiO}}_{2}$-3 and *CoP*/${\mathrm{TiO}}_{2}$-5. EDX spectra also contain peaks of impurities, which show that it is impossible to remove all the impurities from the sample by washing. The % weight of different elements estimated from the spectra depicted in Figure A5 are summarized in Table A1.

### 3.6. Electrochemical Analysis

The electrochemical and energy-storage properties of the synthesized materials were investigated using cyclic voltammetry (CV), electrochemical impedance spectroscopy (EIS), and galvanostatic charge-discharge (GCD) measurements in a three-electrode configuration with 0.5 M $H_{2}{\mathrm{SO}}_{4}$ as the electrolyte. These measurements were performed to evaluate and compare the electrochemical performance of the prepared materials. The specific capacitance values were calculated from the corresponding CV and GCD measurements. Although the present study provides a comparative assessment of the electrochemical behavior of the synthesized composites, electrode-to-electrode reproducibility and statistical variations were not systematically evaluated.

#### 3.6.1. Cyclic Voltammetry

The calculated specific capacitances of ${\mathrm{CoP}/\mathrm{TiO}}_{2}$-1, ${\mathrm{CoP}/\mathrm{TiO}}_{2}$-2, ${\mathrm{CoP}/\mathrm{TiO}}_{2}$-3, ${\mathrm{CoP}/\mathrm{TiO}}_{2}$-4, and ${\mathrm{CoP}/\mathrm{TiO}}_{2}$-5 were 264, 345, 362, 375, and 433 F $g^{-1}$, respectively, indicating a progressive improvement in the electrochemical charge-storage performance across the investigated composition series Figure A7. However, as presented in Table 1, the preparation of these composites involved simultaneous increases in thiophene content (0.1–0.9 mL) and ${\mathrm{TiO}}_{2}$ content (0.04–0.12 g), while the aniline content remained constant at 0.2 mL. Therefore, the observed increase in specific capacitance cannot be attributed exclusively to ${\mathrm{TiO}}_{2}$ incorporation. Instead, the improvement may arise from the combined effects of variations in conducting polymer composition, ${\mathrm{TiO}}_{2}$ content, and the resulting structural and electrochemical characteristics of the hybrid materials. Changes in the relative proportion of aniline and thiophene may influence the redox behavior and charge-storage properties of the polymer matrix, whereas ${\mathrm{TiO}}_{2}$ incorporation may also affect the electrochemical response of the composites. Among the investigated formulations, ${\mathrm{CoP}/\mathrm{TiO}}_{2}$-5 exhibited the highest specific capacitance, demonstrating the most favorable charge-storage performance under the tested conditions. Nevertheless, independently varying the thiophene and ${\mathrm{TiO}}_{2}$ contents would be necessary to determine their individual contributions and establish a definitive composition–performance relationship. Capacitance can be calculated from the cyclic voltammogram by using Equation (1).

(1) $$C=\frac{1}{\nu (V_{f}-V_{i})}\int_{V_{i}}^{V_{f}}I_{m}\;dV$$

where, ν is the scan rate, $\left(V_{f}-V_{i}\right)$ is the potential range, and $I_{m}$ gives the response current. Specific capacitance ($C_{s}$) in terms of F $g^{-1}$, is calculated from Equation (2), as;

(2) $$C_{s}=\frac{1}{\nu m(V_{f}-V_{i})}\int_{V_{i}}^{V_{f}}I_{m}\;dV$$

**Figure A6:**
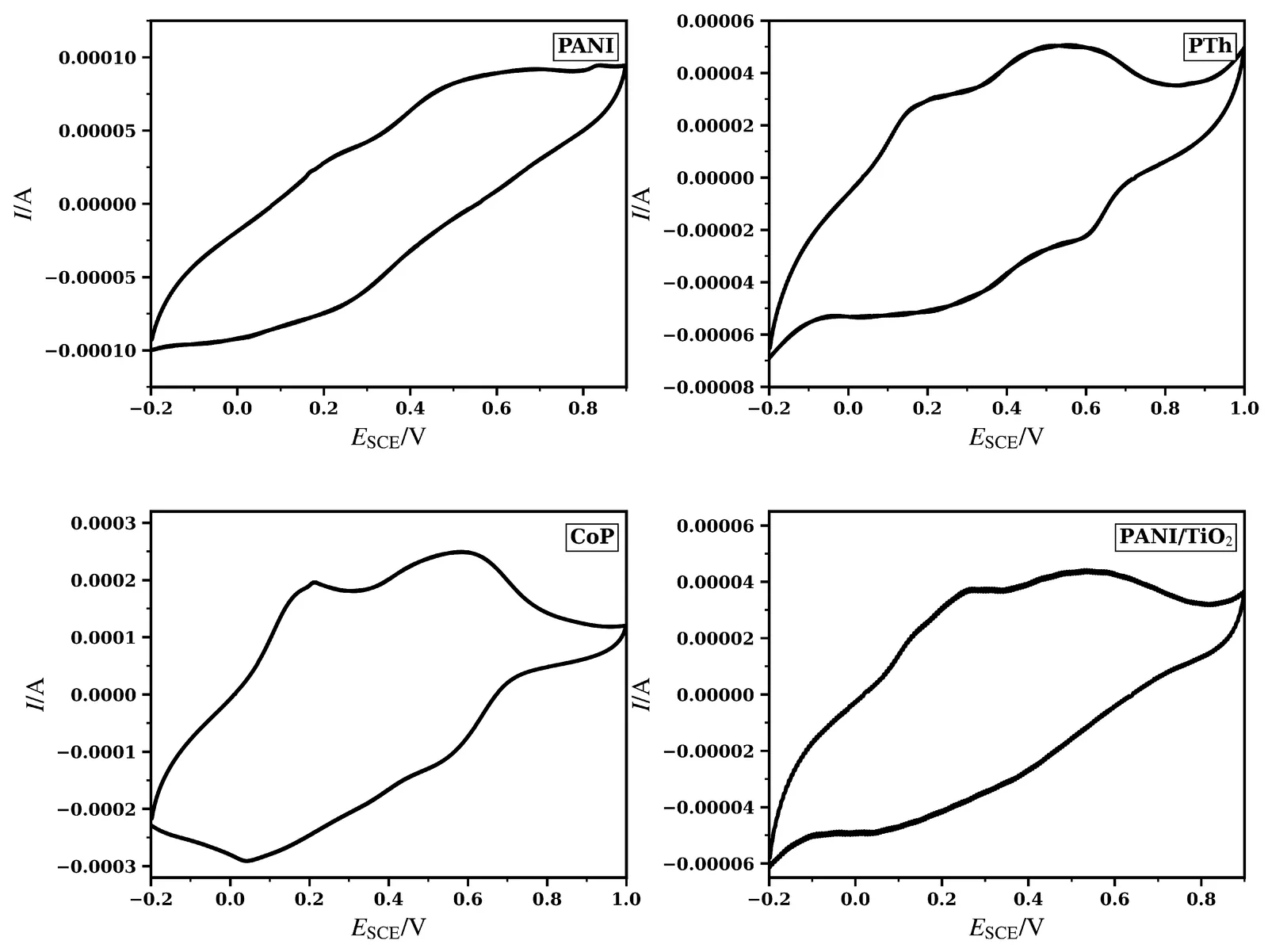
CV curves of PANI, PTh, ${\mathrm{PANI}/\mathrm{TiO}}_{2}$ and CoP in 0.5 M $H_{2}{\mathrm{SO}}_{4}$ solution recorded at scan rate of 40 mV/s.

**Figure A7:**
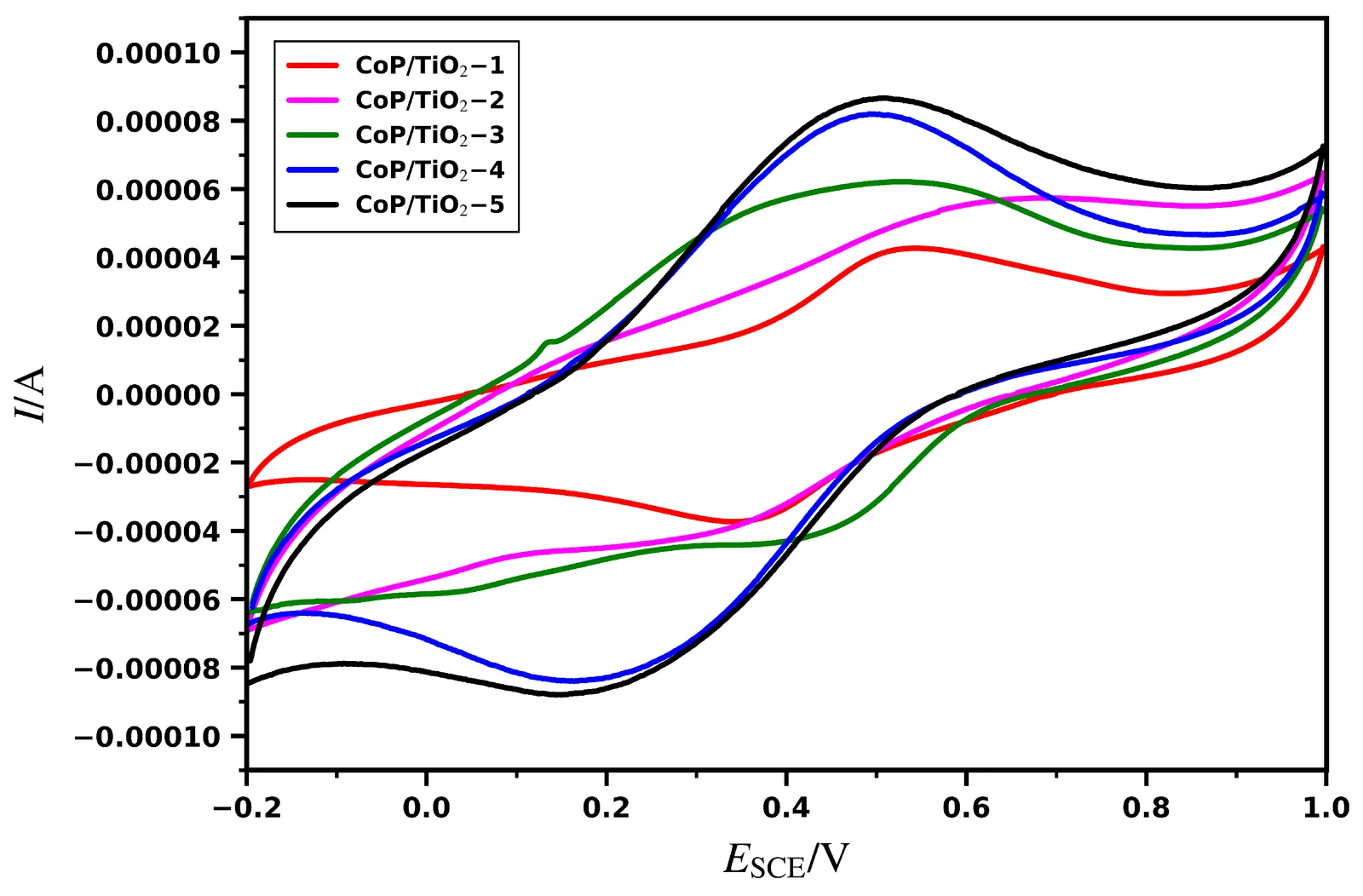
CV curves ${\mathrm{CoP}/\mathrm{TiO}}_{2}$-1 to ${\mathrm{CoP}/\mathrm{TiO}}_{2}$-5 composites in 0.5 M $H_{2}{\mathrm{SO}}_{4}$ solution recorded at scan rate of 40 mV/s.

For the detailed study of the electrochemical properties, the synthesized materials were studied in a three-electrode cell configuration in $H_{2}{\mathrm{SO}}_{4}$ electrolyte. Figure A6 displays the CV curves of PANI, PTh, ${\mathrm{PANI}/\mathrm{TiO}}_{2}$, and *CoP* at 40 mV $s^{-1}$ in 0.5 M $H_{2}{\mathrm{SO}}_{4}$ electrolyte in the potential range of −0.2 to +0.9 V. The CV curves of PANI showed a series of peaks in the oxidation and reduction halves consistent with the redox transition between the semiconducting (leucoemeraldine state) and a conducting emeraldine state of PANI. The CV curve of PTH shows redox peaks due to the oxidation and reduction behavior of PTH. The CV of ${\mathrm{TiO}}_{2}$ recorded at a scan rate of 40 mV $s^{-1}$, as shown in Figure A6, indicates capacitive behavior with a rectangular-shaped curve. The CV curves of ${\mathrm{TiO}}_{2}$ do not change shape as the scan rate increases from 10 to 200 mV $s^{-1}$, representing high-rate capability and high capacitive behavior of the ${\mathrm{TiO}}_{2}$. Since there is no evidence of any faradaic reactions on the rectangular CV curves, the ${\mathrm{TiO}}_{2}$ acts as a pure double-layer capacitor. The CV curve of the CoP in Figure A6 exhibits a pair of redox peaks, ascribed to the anion doping-dedoping processes. Copolymer redox potentials are different from those of the pure PTh and PANI polymers, indicating the formation of a different material. The CV response of CoP differs from those of the individual PANI and PTh electrodes, suggesting changes in electrochemical redox behavior associated with the combined polymer composition. However, these differences cannot be directly attributed to increased specific surface area without quantitative surface-area measurements. The CV curve of the ${\mathrm{PANI}/\mathrm{TiO}}_{2}$ composite electrode in Figure A6 resembles that of PANI. However, the area under the curve for the ${\mathrm{PANI}/\mathrm{TiO}}_{2}$ composite is greater than that of the PANI electrode. The maximum capacitance of the ${\mathrm{TiO}}_{2}$ is only 231 F $g^{-1}$, and the capacitance of the ${\mathrm{PANI}/\mathrm{TiO}}_{2}$ sample is 272 F $g^{-1}$; the deposition of PANI could significantly improve the capacitance performance of the electrodes. The calculated specific capacitances of the synthesized PANI, PTh, ${\mathrm{TiO}}_{2}$, ${\mathrm{PANI}/\mathrm{TiO}}_{2}$ and ${\mathrm{CoP}/\mathrm{TiO}}_{2}$ were 264 F $g^{-1}$, 106 F $g^{-1}$, 231 F $g^{-1}$ and 272 F $g^{-1}$, respectively. Figure A7 shows the CV curves of ${\mathrm{CoP}/\mathrm{TiO}}_{2}$ (${\mathrm{CoP}/\mathrm{TiO}}_{2}$-1–CoP/TiO_2_-5). All the curves display a semi-rectangular shape with a couple of redox peaks. The calculated specific capacitances of *CoP*/${\mathrm{TiO}}_{2}$-1, *CoP*/${\mathrm{TiO}}_{2}$-2, ${\mathrm{CoP}/\mathrm{TiO}}_{2}$-3, *CoP*/${\mathrm{TiO}}_{2}$-4 and ${\mathrm{CoP}/\mathrm{TiO}}_{2}$-5 were 264 F $g^{-1}$, 345 F $g^{-1}$, 362 F $g^{-1}$, 375 F $g^{-1}$ and 433 F $g^{-1}$, respectively.

**Figure 4:**
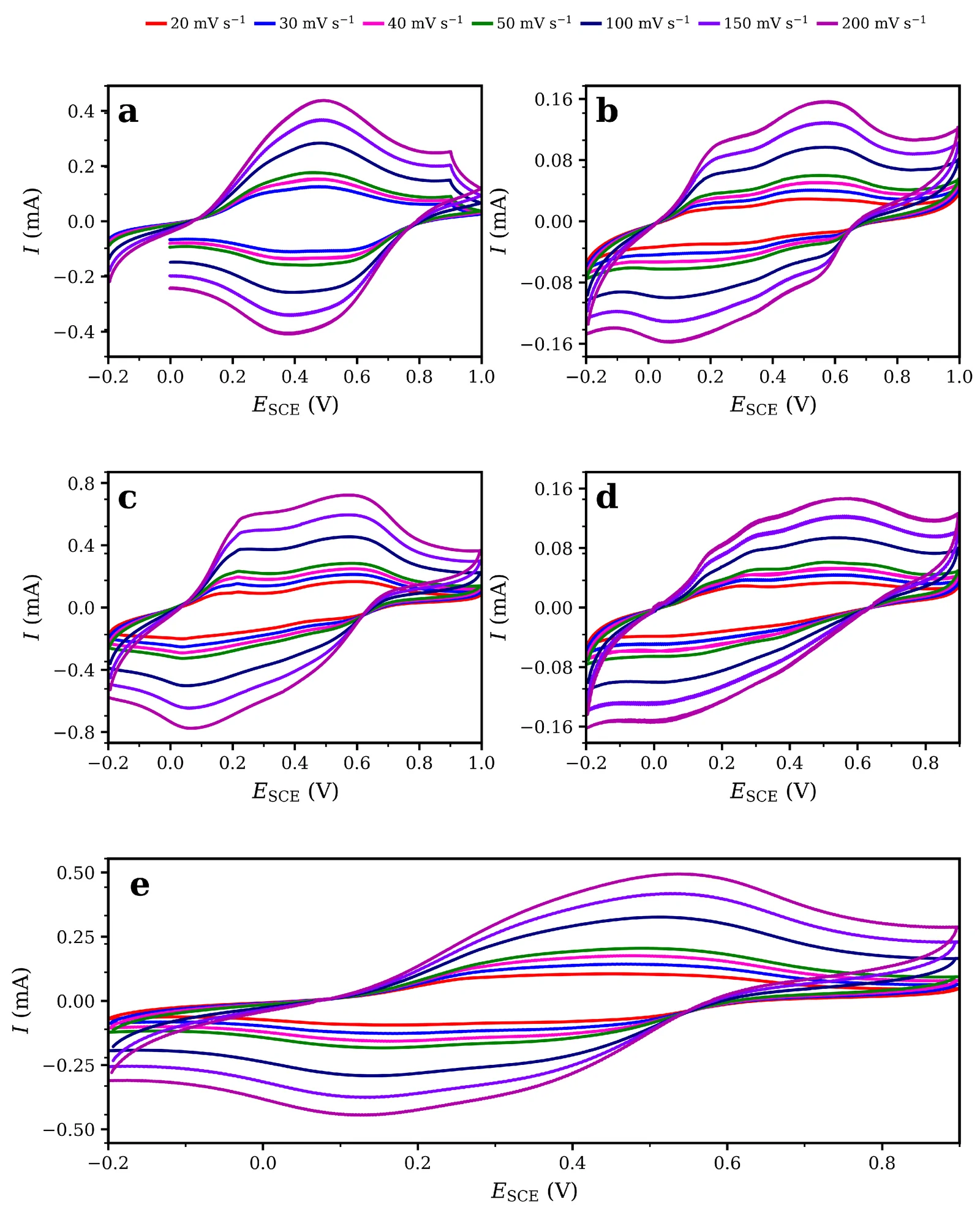
CV curves recorded at different scan rates in 0.5 M $H_{2}{\mathrm{SO}}_{4}$ solution for (**a**) PANI; (**b**) PTh; (**c**) CoP; (**d**) ${\mathrm{PANI}/\mathrm{TiO}}_{2}$; and (**e**) ${\mathrm{CoP}/\mathrm{TiO}}_{2}$-5.

Figure 4 represents the CV curves of PANI, PTh, ${\mathrm{PANI}/\mathrm{TiO}}_{2}$, *CoP* and *CoP*/${\mathrm{TiO}}_{2}$-5 at different scan rates. The CV curves of the ${\mathrm{PANI}/\mathrm{TiO}}_{2}$ composite electrode show the mechanism of proton transfer, which is slower at a higher scan rate, due to degradation or saturation of the protons inside the electrode during the redox process. This results mostly in improved ionic resistivity, contributing to a decrease in electrode capacitance. It is also assumed that the real capacitance obtained at the slow scan speeds is comparable to that of the maximum usage of the electrode material. At 10 mV $s^{-1}$ scan rate, the maximum specific capacitance of PANI and ${\mathrm{PANI}/\mathrm{TiO}}_{2}$ composite electrodes are $264\;{F\;g}^{-1}$ and $272\;{F\;g}^{-1}$, respectively. Here, because of counter-ions intercalation/deintercalation, ${\mathrm{TiO}}_{2}$ nanocomposites may provide a shield against volume shifts of PANI during stabilization. Furthermore, ${\mathrm{TiO}}_{2}$ may avoid breakage of the PANI chain, resulting in a stable chain structure during the charging and discharging process. The synergistic influence of ${\mathrm{TiO}}_{2}$ together with the conducting PANI, therefore, plays a significant role not only in diffusion of the electrolyte ions but also in decreasing mechanical degradation. ${\mathrm{TiO}}_{2}$ increases the stability of PANI electrode in ${\mathrm{PANI}/\mathrm{TiO}}_{2}$ composite electrode, resulting a high specific capacitance. CV curves of all samples at different scan rates show that as the scan rate increases, the sample peaks become more and more prominent and start merging into each other and fully merge at a scan rate of 200 mV $s^{-1}$. The value of capacitance decreases with increasing scan rate. This is due to the part of the electrode surface that contributes at high charging/discharging rates, which decreases the specific capacitance value with increasing scan rate.

**Figure 5:**
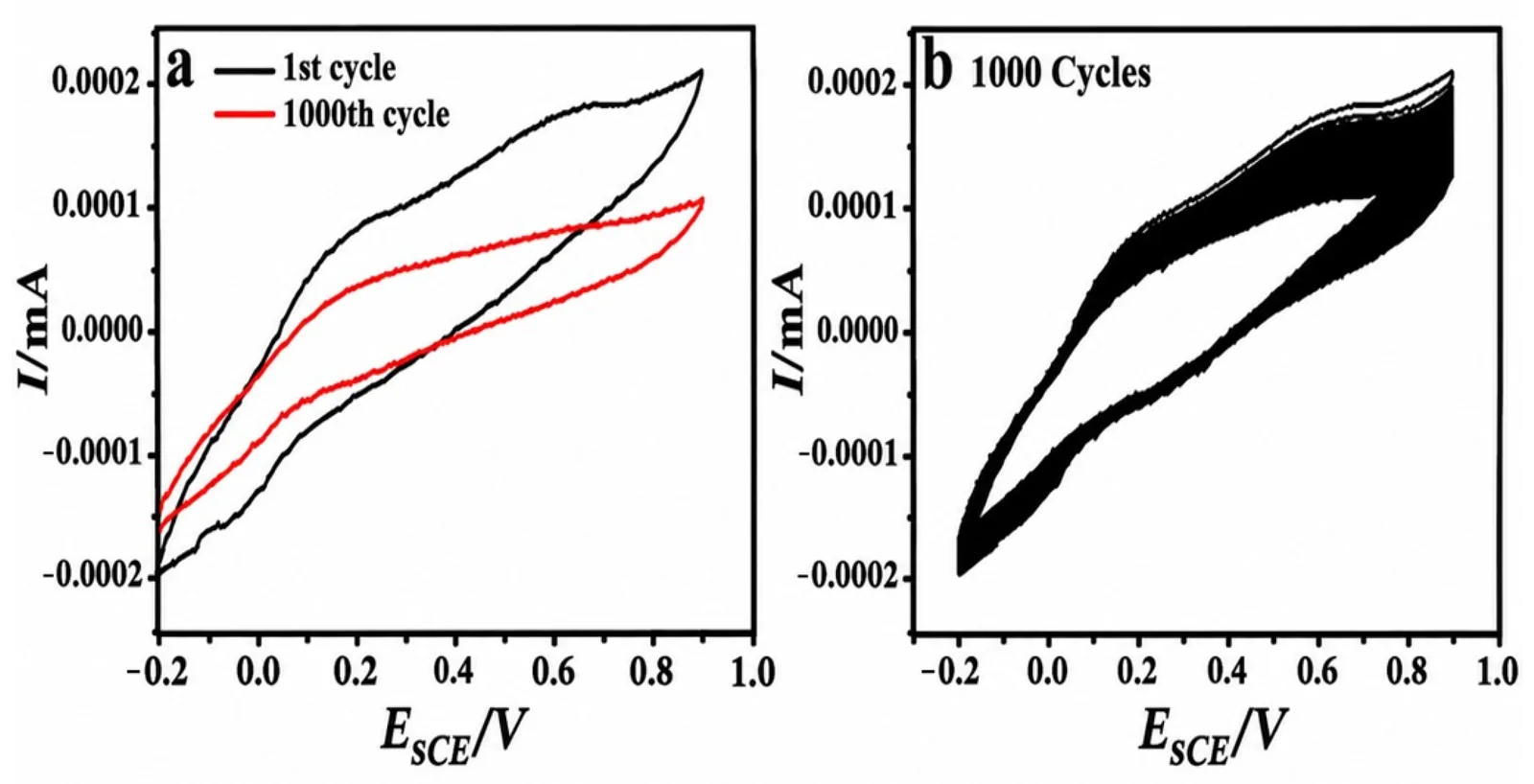
CV curves of ${\mathrm{CoP}/\mathrm{TiO}}_{2}$ in 0.5 M $H_{2}{\mathrm{SO}}_{4}$ solution recorded at scan rate of 40 mV/s (**a**) 1st and 1000th cycle and (**b**) 1000 consecutive cycles.

The cycling stability of the optimized ${\mathrm{CoP}/\mathrm{TiO}}_{2}$-5 electrode was evaluated through 1000 consecutive cyclic voltammetry cycles in 0.5 M $H_{2}{\mathrm{SO}}_{4}$ electrolyte, as illustrated in Figure 5b. The electrode retained approximately 61% of its initial specific capacitance after 1000 cycles, corresponding to a capacitance loss of approximately 39%. This reduction indicates a substantial decrease in electrochemical charge-storage performance during repeated cycling. The observed capacitance decay may be associated with changes in the electrochemical activity of the conducting polymer matrix during successive doping–dedoping processes; however, the specific degradation mechanism cannot be established from the available measurements. Although the ${\mathrm{CoP}/\mathrm{TiO}}_{2}$-5 electrode exhibited higher initial capacitance than the investigated reference materials, the present results do not independently demonstrate improved cycling durability arising from ${\mathrm{TiO}}_{2}$ incorporation. In particular, comparative cycling retention measurements for PANI, CoP, and ${\mathrm{PANI}/\mathrm{TiO}}_{2}$ electrodes are necessary to establish whether the hybrid architecture provides improved long-term electrochemical stability. Therefore, the present cycling results highlight a limitation of the optimized composite and indicate the need for further investigation of its degradation behavior and long-term operational performance.

#### 3.6.2. Galvanostatic Charge/Discharge (GCD) Measurements

**Figure 6:**
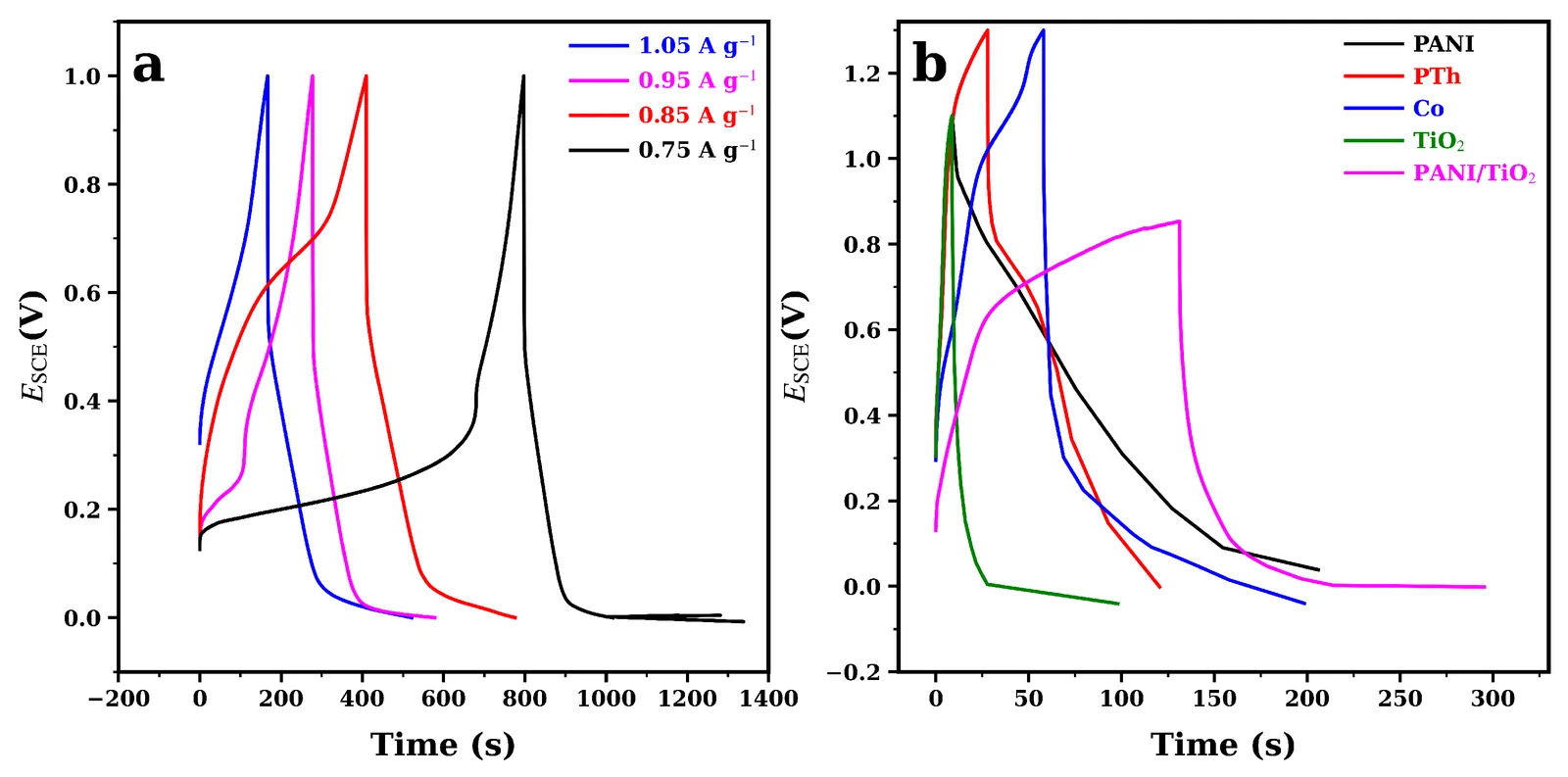
(**a**) Charge discharge curves of PANI, PTh, Co, ${\mathrm{TiO}}_{2}$, ${\mathrm{PANI}/\mathrm{TiO}}_{2}$ and ${\mathrm{CoP}/\mathrm{TiO}}_{2}$ measured at $0.75\;{A\;g}^{-1}$ in 0.5 M $H_{2}{\mathrm{SO}}_{4}$ solution. (**b**) GCD curve of ${\mathrm{CoP}/\mathrm{TiO}}_{2}$-5 at different current densities.

The GCD is considered a more reliable technique for capacitance measurement. The GCD test was employed for all synthesized samples in 0.5 M electrolyte ($H_{2}{\mathrm{SO}}_{4}$ solution) at $0.75\;{A\;g}^{-1}$ within the potential range of 0.0 V to 0.8 V, and the data are displayed below in Figure 6b.

The specific capacitance from GCD curves can be calculated using the following Equation (3).

(3) $$Csp=\frac{I.\Delta t}{m.\Delta V}$$

where,

I=current (A),

Δt=discharge time (s)

m=mass of electrode material (g),

ΔV=applied potential (Volts)

The calculated specific capacitance of PANI, PTh, ${\mathrm{TiO}}_{2}$, *CoP* and ${\mathrm{PANI}/\mathrm{TiO}}_{2}$ at the 0.75 A $g^{-1}$ discharge current density was 198 F $g^{-1}$, 56 F $g^{-1}$, 65 F $g^{-1}$, 85 F $g^{-1}$ and 216 F $g^{-1}$, respectively. The galvanostatic charge–discharge measurements demonstrate that the ${\mathrm{CoP}/\mathrm{TiO}}_{2}$-5 electrode exhibits a longer discharge duration and higher specific capacitance than the investigated reference electrodes under the reported testing conditions. At a current density of 0.75 A $g^{-1}$, the ${\mathrm{CoP}/\mathrm{TiO}}_{2}$-5 composite delivered a specific capacitance of 414 F $g^{-1}$, compared with 198 F $g^{-1}$ for PANI, 56 F $g^{-1}$ for PTh, 65 F $g^{-1}$ for ${\mathrm{TiO}}_{2}$, 85 F $g^{-1}$ for CoP, and 216 F $g^{-1}$ for ${\mathrm{PANI}/\mathrm{TiO}}_{2}$. The enhanced electrochemical response may be associated with the combined effects of conducting polymer composition, ${\mathrm{TiO}}_{2}$ incorporation, and the redox-active characteristics of the hybrid material. However, the higher capacitance cannot be directly attributed to increased porosity or accessible surface area because quantitative BET surface-area and pore-size-distribution measurements were not performed. Although SEM analysis reveals morphological differences among the prepared composites, these observations alone are insufficient to establish enhanced electrolyte accessibility or ion transport. Therefore, the improved capacitance is discussed primarily in terms of the experimentally observed electrochemical behavior, while the specific contributions of surface area and pore structure remain to be determined, as shown in Figure 6a.

To further assess the electrochemical advantage of the ternary hybrid formulation, the specific capacitance of the optimized ${\mathrm{CoP}/\mathrm{TiO}}_{2}$-5 electrode was compared with those of the individual components and binary composite materials investigated under the same reported experimental conditions. At a current density of 0.75 A $g^{-1}$, the ${\mathrm{CoP}/\mathrm{TiO}}_{2}$-5 electrode exhibited a specific capacitance of 414 F $g^{-1}$, considerably higher than those of pristine PANI (198 F $g^{-1}$), PTh (56 F $g^{-1}$), ${\mathrm{TiO}}_{2}$ (65 F $g^{-1}$), CoP (85 F $g^{-1}$), and ${\mathrm{PANI}/\mathrm{TiO}}_{2}$ (216 F $g^{-1}$). This comparative improvement indicates that the ${\mathrm{CoP}/\mathrm{TiO}}_{2}$-5 formulation exhibits more favorable electrochemical charge-storage characteristics than the reference electrodes under the investigated conditions. The structural and spectroscopic characterization results further support the incorporation of anatase ${\mathrm{TiO}}_{2}$ within the PANI/PTh-based matrix. However, the observed capacitance enhancement may reflect the combined influence of polymer composition, ${\mathrm{TiO}}_{2}$ incorporation, and electrode morphology. Since the thiophene and ${\mathrm{TiO}}_{2}$ quantities were simultaneously varied across the composition series, their individual contributions cannot be independently resolved from the present results. Moreover, although interactions between the polymer matrix and ${\mathrm{TiO}}_{2}$ may contribute to the observed electrochemical response, direct confirmation of a specific interfacial charge-transfer mechanism requires additional experimental evidence. Therefore, the principal contribution of this investigation is the preparation and comparative electrochemical assessment of PANI/PTh–anatase ${\mathrm{TiO}}_{2}$ formulations, together with the identification of a composition exhibiting enhanced capacitance relative to the investigated reference materials.

The GCD curve of ${\mathrm{CoP}/\mathrm{TiO}}_{2}$-5, Figure 6b, was recorded at different current densities in order to evaluate the effect of current density on capacitance. The measured capacitance of ${\mathrm{CoP}/\mathrm{TiO}}_{2}$-5 at current density 0.75 A $g^{-1}$, 0.85 A $g^{-1}$, 0.95 A $g^{-1}$ and 1.05 A $g^{-1}$ are to be 414 F $g^{-1}$, 372 F $g^{-1}$, 346 F $g^{-1}$ and 316 F $g^{-1}$, respectively. The result shows that current density increases as the discharge time decreases, which is due to the non-uniform distribution of current in the porous matrix. It means that the ions easily diffuse into the microstructure (porous material) at low current density, and at high current density the ions will not completely diffuse into the active materials due to the high steric hindrance and limitation of ion accessibility to the inner surface. GCD results also confirm that at higher current density, the charge-discharge time decreases; hence, capacitance also decreases.

The electrochemical performance of the synthesized ${\mathrm{CoP}/\mathrm{TiO}}_{2}$ composites can also be considered in relation to recently reported conducting polymer–metal oxide electrodes. The optimized ${\mathrm{CoP}/\mathrm{TiO}}_{2}$-5 electrode exhibited a specific capacitance of 414 F $g^{-1}$ at 0.75 A $g^{-1}$, demonstrating a higher capacitance than the individual polymer components and the ${\mathrm{PANI}/\mathrm{TiO}}_{2}$ electrode investigated under the same experimental conditions. In comparison, Hacinecipoğlu et al. [62] reported a capacitance of 294 F $g^{-1}$ at 10 mV $s^{-1}$ for a PbO/poly(aniline-co-thiophene) electrode, whereas Kumar et al. [64] reported 271.3 F $g^{-1}$ at 1 A $g^{-1}$ for a ${\mathrm{PTh/MoO}}_{3}{\mathrm{/TiO}}_{2}$ ternary composite. Furthermore, Fazal et al. [63] demonstrated a specific capacity of 925 C $g^{-1}$ at 1 A $g^{-1}$ for a ${\mathrm{TiO}}_{2}$/PANI composite. Although these investigations demonstrate the electrochemical potential of conducting polymer–metal oxide architectures, direct quantitative ranking is complicated by differences in electrolyte composition, current density, electrode configuration, and charge-storage metrics. The present results, therefore, establish an improvement in capacitance relative to the reference materials evaluated within this study, while further investigations are required to clarify the specific interfacial contributions and long-term electrochemical stability of the ${\mathrm{CoP}/\mathrm{TiO}}_{2}$ system.

#### 3.6.3. Electrochemical Impedance Spectroscopy (EIS) Measurements

**Figure 7:**
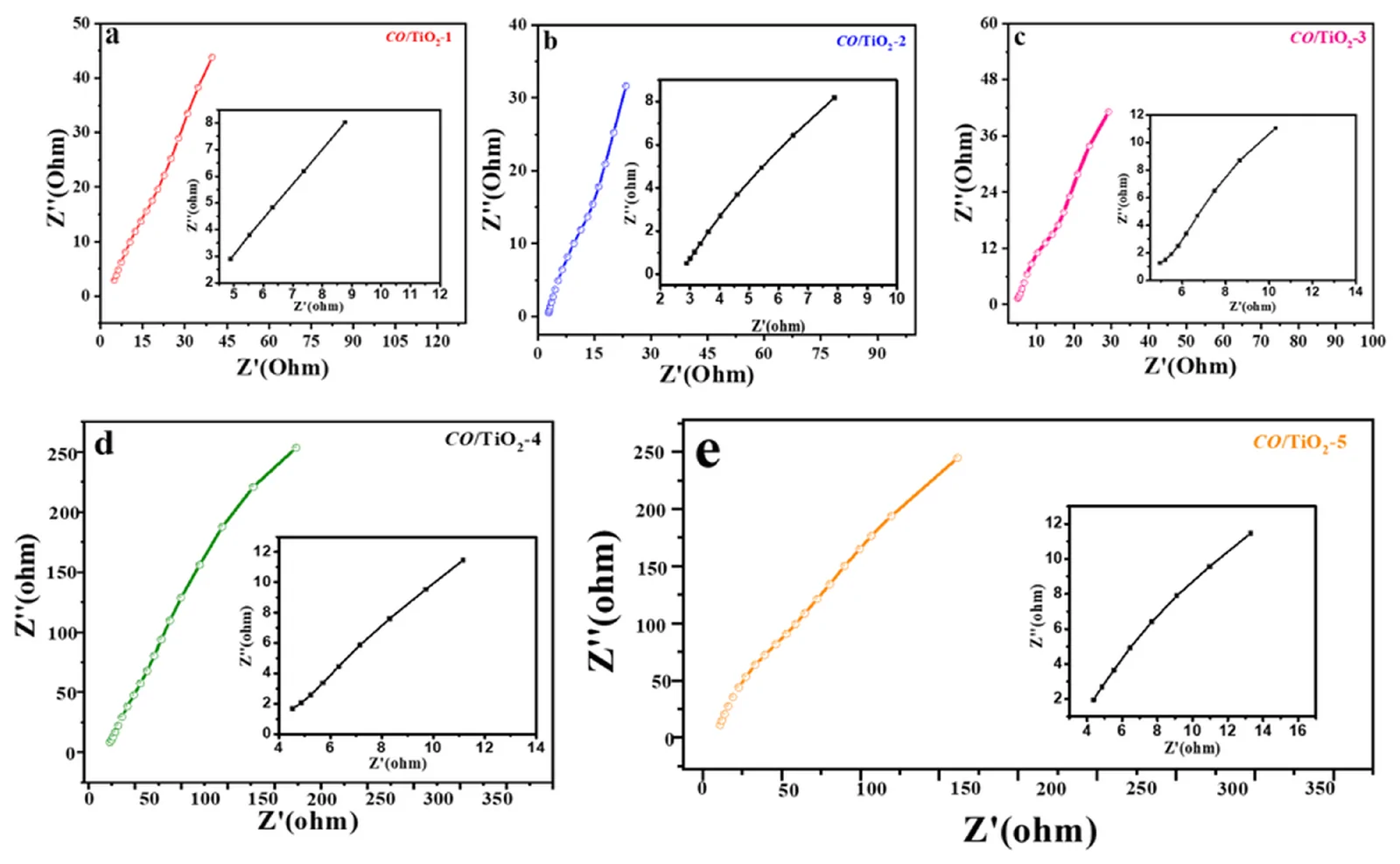
Nyquist plots of (**a**) ${\mathrm{CoP}/\mathrm{TiO}}_{2}$-1, (**b**) ${\mathrm{CoP}/\mathrm{TiO}}_{2}$-2, (**c**) ${\mathrm{CoP}/\mathrm{TiO}}_{2}$-3, (**d**) ${\mathrm{CoP}/\mathrm{TiO}}_{2}$-4 and (**e**) ${\mathrm{CoP}/\mathrm{TiO}}_{2}$-5 in 0.5 M $H_{2}{\mathrm{SO}}_{4}$.

**Figure A8:**
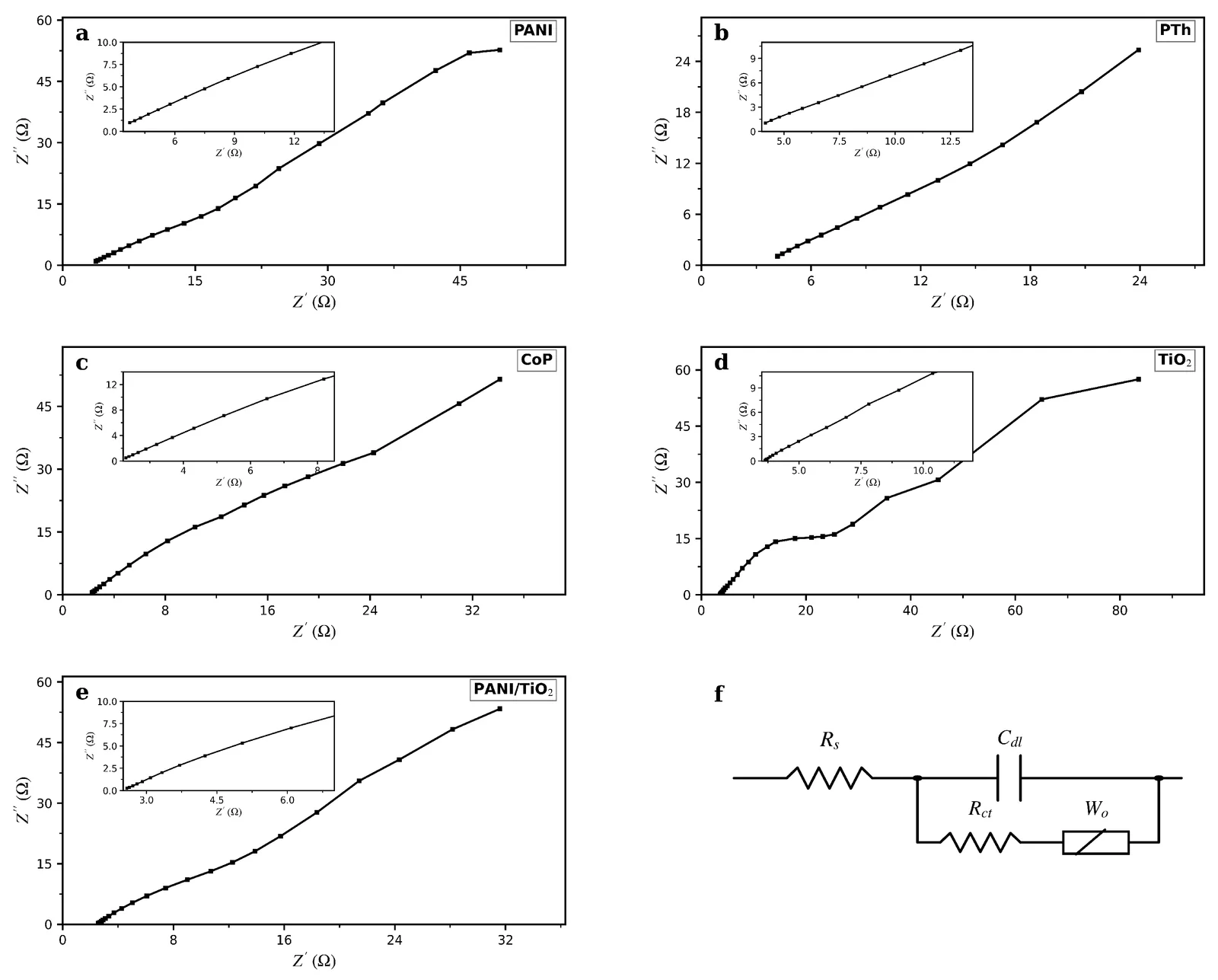
Nyquist plots of (**a**) PANI, (**b**) PTh, (**c**) CoP, (**d**) ${\mathrm{TiO}}_{2}$, and (**e**) ${\mathrm{PANI}/\mathrm{TiO}}_{2}$, in 0.5 M $H_{2}{\mathrm{SO}}_{4}$. (**f**) EIS fittings.

EIS is the most powerful and promising technique for the evaluation of interfacial properties like capacitance, reaction mechanism and ionic and electronic influences of surface-modified electrode materials. Figure A8 shows the Nyquist plots of (a) PANI, (b) PTh, (c) CoP, (d) ${\mathrm{TiO}}_{2}$ and (e) ${\mathrm{PANI}/\mathrm{TiO}}_{2}$ composite electrodes. All the plots exhibit common features of semicircles in the high-frequency region and straight lines in the medium- to low-frequency region. The resistance on the x-axis from 0 to the start point of the semicircle represents the solution resistance, which is at the interface of the electrolyte solution and the active material of the electrode. All electrodes show nearly identical values of solution resistance regardless of the nature of the material because the same electrolyte was used during analysis. The semicircle corresponds to the bulk resistance of the active material in parallel with the interfacial charge transfer. PANI and ${\mathrm{PANI}/\mathrm{TiO}}_{2}$ composite electrodes have smaller semicircles (see the expanded portion), corresponding to the lower charge transfer resistance of these electrodes. The straight lines observed in the electrodes are due to the diffusion of ions and/or non-uniform concentration profiles of the charged species in the film. The slope of the lines at the end of the semicircle determines whether the behavior of the electrode is close to that of the capacitor or a battery. In the case of the capacitor, the slope of the line is close to $90^{\circ }$, while in the case of a battery, the slope is around $45^{\circ }$ due to Warburg diffusion. All samples in Figure A8 show a line with a slope between $90^{\circ }$ and $45^{\circ }$, thus indicating the characteristic of these electrodes to be like a pseudocapacitor. Figure 7 presents the Nyquist plots of the ${\mathrm{CoP}/\mathrm{TiO}}_{2}$-1 to ${\mathrm{CoP}/\mathrm{TiO}}_{2}$-5 composite electrodes measured in 0.5 M $H_{2}{\mathrm{SO}}_{4}$ electrolyte. The impedance spectra exhibit high-frequency features and inclined responses in the lower-frequency region, reflecting contributions from resistive, capacitive, and ion-transport processes within the electrode systems. Differences in the impedance profiles among the synthesized composites suggest that their electrochemical responses are influenced by the formulation composition. The observed changes may be associated with variations in polymer composition, ${\mathrm{TiO}}_{2}$ incorporation, and the resulting electrode characteristics. Nevertheless, qualitative differences in the Nyquist plots alone do not establish a specific interfacial charge-transfer mechanism or demonstrate improved electrolyte accessibility. Furthermore, because the thiophene and ${\mathrm{TiO}}_{2}$ contents were simultaneously varied, the individual contributions of these components to the impedance response cannot be independently determined. Quantitative equivalent-circuit fitting is required to separate the solution resistance, charge-transfer resistance, nonideal capacitive behavior, and diffusion-related impedance contributions. Therefore, the present impedance results are interpreted as evidence of composition-dependent electrochemical behavior rather than direct confirmation of enhanced interfacial charge transfer or suppression of polymer degradation. Differences in the Nyquist plots were observed among the ${\mathrm{CoP}/\mathrm{TiO}}_{2}$ formulations, suggesting composition-dependent variations in their electrochemical impedance characteristics. However, because both thiophene and ${\mathrm{TiO}}_{2}$ contents were simultaneously varied, these differences cannot be assigned exclusively to increasing ${\mathrm{TiO}}_{2}$ content. Quantitative equivalent-circuit analysis would be required to establish changes in the individual resistance and capacitive contributions.

## 4. Conclusions

PANI, PTh, Poly(aniline-co-thiophene) and their composites with ${\mathrm{TiO}}_{2}$ were synthesized chemically by inverse emulsion polymerization in the search for new hybrid materials for energy storage. After preparation, the composite materials were investigated with different spectroscopic and microscopic techniques for structural and morphological characterization and subsequently tested for electrochemical properties and energy storage capacity. The Poly(aniline-co-thiophene)/${\mathrm{TiO}}_{2}$. (${\mathrm{CoP}/\mathrm{TiO}}_{2}$) composite shows comparatively better capacitance and energy storage capability as compared with its constituents. ${\mathrm{CoP}/\mathrm{TiO}}_{2}$ composite has a specific capacitance of 433 F $g^{-1}$ measured from CV at a scan rate of 10 mV/s and 414 F $g^{-1}$ calculated from GCD at 0.75 A $g^{-1}$ current density. The optimized ${\mathrm{CoP}/\mathrm{TiO}}_{2}$-5 composite exhibited a specific capacitance of 433 F $g^{-1}$ from CV measurements and 414 F $g^{-1}$ from GCD measurements at a current density of 0.75 A $g^{-1}$. However, the electrode retained approximately 61% of its initial capacitance after 1000 consecutive cycles, revealing a considerable decline in charge-storage performance during repeated cycling. Although the composite demonstrated enhanced initial capacitance relative to the investigated reference materials, its cycling durability remains a limitation. Further investigations involving comparative cycling measurements, structural characterization before and after cycling, and longer-term electrochemical stability assessments are necessary to clarify the degradation mechanism and evaluate its suitability for practical supercapacitor applications.

## Figures and Tables

**Table 1:** Composition of synthesized *CoP*/${\mathrm{TiO}}_{2}$ composites.

Composite	Amount of Aniline (mL)	Amount of Thiophene (mL)	Amount of ${\mathrm{TiO}}_{2}$ (g)
${\mathrm{CoP}/\mathrm{TiO}}_{2}$-1	0.2	0.1	0.04
${\mathrm{CoP}/\mathrm{TiO}}_{2}$-2	0.2	0.3	0.06
${\mathrm{CoP}/\mathrm{TiO}}_{2}$-3	0.2	0.5	0.08
${\mathrm{CoP}/\mathrm{TiO}}_{2}$-4	0.2	0.7	0.10
${\mathrm{CoP}/\mathrm{TiO}}_{2}$-5	0.2	0.9	0.12

**Figure A4:**
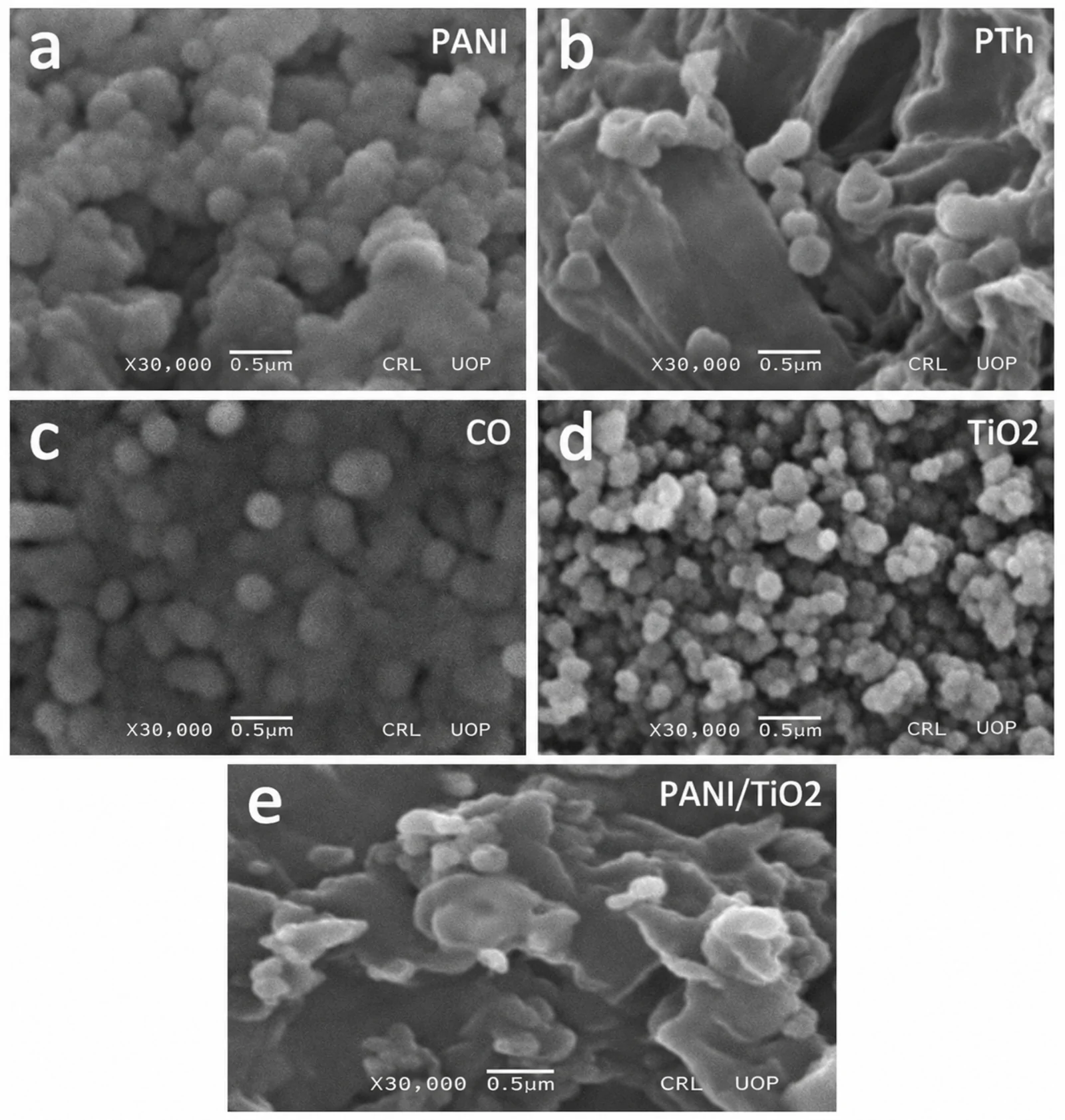
SEM images of (**a**) PANI, (**b**) PTh, (**c**) *CoP*, (**d**) ${\mathrm{TiO}}_{2}$ and (**e**) ${\mathrm{PANI}/\mathrm{TiO}}_{2}$.

## Data Availability

The original contributions presented in this study are included in the article. Further inquiries can be directed to the corresponding authors.

## Appendix A. Supplementary Experimental Information

### Characterization

UV-Vis spectra of the prepared materials were recorded using a UV/Vis spectrophotometer (Shimadzu, Kyoto, Japan). The crystallinity of particles was determined with an X-ray diffractometer (JEOL JDX-3532, Akishima, Tokyo, Japan) under Cu Kα radiations (λ=1.5405 Å). Scanning electron microscope (SEM, JEOL JSM-6490, Akishima, Tokyo, Japan), was used to determine the surface morphology of the synthesized samples. The Fourier Transform Infrared Spectroscopy (FTIR) measurements were performed with an IR Affinity-1 spectrophotometer (Shimadzu, Kyoto, Japan.) in the wavelength range of $500{\mathrm{cm}}^{-1}$–$4000{\mathrm{cm}}^{-1}$. The elemental composition of the samples was determined with energy-dispersive X-ray spectroscopy (EDX).

Electrochemical measurements in 0.5 M $H_{2}{\mathrm{SO}}_{4}$ were carried out using a Potentiostat/galvanostat (Gamry Instruments-600 ZRA, Warminster, PA, USA). A three-electrode system having the working electrode (composite-coated gold electrode), a reference electrode (calomel electrode) and a counter electrode (gold electrode) was used. Chloroform was used to make solutions of the PANI, PTh, ${\mathrm{TiO}}_{2}$, ${\mathrm{PANI}/\mathrm{TiO}}_{2}$, CoP and ${\mathrm{CoP}/\mathrm{TiO}}_{2}$, which were then coated by the drop-casting process on the gold electrode surface. CV, GCD and EIS analyses were performed to examine the capacitive performance and cyclic stability of ${\mathrm{TiO}}_{2}$, PANI, PTh, and the composites ${\mathrm{PANI}/\mathrm{TiO}}_{2}$, CoP and ${\mathrm{CoP}/\mathrm{TiO}}_{2}$. The electrochemical performance of the synthesized PANI, PTh, ${\mathrm{TiO}}_{2}$, ${\mathrm{PANI}/\mathrm{TiO}}_{2}$, CoP, and ${\mathrm{CoP}/\mathrm{TiO}}_{2}$ composites was investigated using a Gamry potentiostat/galvanostat (Reference 600 ZRA, Warminster, PA, USA) in a conventional three-electrode configuration containing 0.5 M $H_{2}{\mathrm{SO}}_{4}$ as the electrolyte. A composite-coated gold electrode was employed as the working electrode, while a saturated calomel electrode (SCE) and a gold electrode served as the reference and counter electrodes, respectively. The synthesized ${\mathrm{CoP}/\mathrm{TiO}}_{2}$ composite was dispersed in a small amount of N-methyl-2-pyrrolidone (NMP) to prepare a coating suspension, which was subsequently deposited onto a gold (Au) sheet working electrode using the drop-casting method. Approximately 2.0 mg of the active material was deposited on each side of the Au sheet, resulting in a total active-material loading of approximately 4.0 mg per electrode. The coating was performed sequentially, with the first coated surface allowed to dry at room temperature for several hours before applying the suspension to the opposite side. Following deposition, the coated electrode was dried at room temperature before electrochemical measurements. The coating thickness was not experimentally determined.

**Table A1:** Weight percentages of the elements estimated from the EDX spectra.

Element	PANI	PTh	${\mathrm{TiO}}_{2}$	${\mathrm{PANI}/\mathrm{TiO}}_{2}$	CoP	${\mathrm{CoP}/\mathrm{TiO}}_{2}$-1	${\mathrm{CoP}/\mathrm{TiO}}_{2}$-2	${\mathrm{CoP}/\mathrm{TiO}}_{2}$-3	${\mathrm{CoP}/\mathrm{TiO}}_{2}$-5
	Weight%	Weight%	Weight%	Weight%	Weight%	Weight%	Weight%	Weight%	Weight%
C	71.71	31.74	-	17.22	63.43	66.15	10.13	32.04	67.26
N	6.29		-	0.95	6.79	4.72	-	-	3.83
O	6.31	4.19	11.01	14.58	6.27	7.06	16.22	9.54	5.50
S	7.21	48.66	-	10.82	7.14	9.60	1.10	1.06	4.16
Cl	7.56	8.76	-	1.46	7.59	8.24	1.97	8.55	17.06
Ca	0.93	-	-	9.06	7.38	2.25	7.33	0.87	0.35
Si	-	0.54	-	0.28	-	0.21	0.17	0.34	0.25
Fe	-	6.12	-	-	-	-	-	-	-
Ti	-	-	88.99	45.04	-	1.52	62.91	47.60	1.01
Mg	-	-	-	0.39	-	0.24	0.16	-	-
Al	-	-	-	0.19	-	-	-	-	-
F	-	-	-	-	1.40	-	-	-	-
